# SENP6‐Mediated deSUMOylation of Nrf2 Exacerbates Neuronal Oxidative Stress Following Cerebral Ischemia and Reperfusion Injury

**DOI:** 10.1002/advs.202410410

**Published:** 2024-12-24

**Authors:** Qian Xia, Mengxin Que, Gaofeng Zhan, Longqing Zhang, Xue Zhang, Yilin Zhao, Huijuan Zhou, Lu Zheng, Meng Mao, Xing Li

**Affiliations:** ^1^ Department of Anesthesiology and Pain Medicine Hubei Key Laboratory of Geriatric Anesthesia and Perioperative Brain Health, and Wuhan Clinical Research Center for Geriatric Anesthesia Tongji Hospital Tongji Medical College Huazhong University of Science and Technology Wuhan 430030 China; ^2^ Department of Neurobiology School of Basic Medicine Tongji Medical College Huazhong University of Science and Technology Wuhan 430030 China; ^3^ Department of Transfusion The First Affiliated Hospital of Zhengzhou University Zhengzhou 450000 China; ^4^ Department of Anesthesiology and Perioperative Medicine Zhengzhou Central Hospital Affiliated to Zhengzhou University Zhengzhou 450007 China

**Keywords:** ischemic stroke, membrane‐permeable peptide, Nrf2, oxidative stress, SENP6

## Abstract

Oxidative stress is believed to play critical pathophysiological roles in ischemic brain injury, and the nuclear factor erythroid 2‐related factor 2 (Nrf2) signaling pathway is recognized as the most crucial endogenous antioxidant stress damage route. Some research have demonstrated that Nrf2 play critical roles in oxidative stress after ischemic stroke, but the underlying mechanism are not fully elucidated. This study reveals that Nrf2 is modified by SUMOylation and identifies Sentrin/SUMO‐specific protease 6 (SENP6) as a negative regulator of Nrf2 SUMOylation. Notably, SENP6 binds to and mediates the deSUMOylation of Nrf2, which in turn inhibits antioxidant response by enhancing ubiquitination‐dependent degradation of Nrf2, thereby reducing its transcriptional activity, inducing oxidative stress and aggravating neuronal apoptosis after ischemic stroke. Additionally, blocking the interaction between SENP6 and Nrf2 with a cell membrane‐permeable peptide (Tat‐Nrf2) preserves the SUMOylation of Nrf2, effectively attenuates oxidative stress, and rescues neurological functions in mice subjected to ischemic stroke. Furthermore, no toxicity is observed when high doses Tat‐Nrf2 are injected into nonischemic mice. Collectively, this study uncovers a previously unidentified mechanism whereby SUMOylation of Nrf2 regulates oxidative stress and strongly indicates that interventions targeting SENP6 or its interaction with Nrf2 may provide therapeutic benefits for ischemic stroke.

## Graphical abstract

This study uncovers a previously unrecognized role for SENP6‐mediated deSUMOylation of Nrf2 in neuronal oxidative stress after ischemic stroke. Mechanistically, SENP6 binds to and mediates deSUMOylation of Nrf2, which in turn inhibits the antioxidant response by enhancing ubiquitination‐dependent degradation of Nrf2, thereby inducing oxidative stress and exacerbating neuronal damage, highlighting the SENP6‐Nrf2 axis as a promising therapeutic target for ischemic stroke.

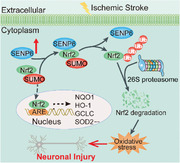

## 1 Introduction

Ischemic stroke, which causes a rapid decrease in cerebral flow, leads to neuronal injury and neurological impairment, which may worsen following recanalization therapy.^[^ ^1^ ^]^ The molecular process of ischemic stroke is exceedingly complex and not fully investigated. Multiple pathophysiological mechanisms are implicated in cerebral ischemia, such as neuronal cell energy metabolism problems, excitotoxicity and acidosis, inflammation, apoptosis, and oxidative stress.^[^ ^2^ ^]^ In particular, because of the excessive formation of reactive oxygen species (ROS) and inadequate ROS clearance, oxidative stress might result in largely negative consequences during the development of cerebral ischemia‐reperfusion (I/R) injury.^[^ ^3^ ^]^ As a result, affected brain tissue undergoes autophagy, necrosis, or apoptosis, which can result in temporary or permanent neurological deficits.^[^ ^4^ ^]^ Redox disturbance has been implicated in exacerbating clinical outcomes in individuals with acute ischemic stroke, but the molecular mechanisms remain largely unknown.^[^ ^5^ ^]^ The obscurity surrounding the processes involved in oxidative stress following cerebral I/R injury may be to blame for the ineffectiveness of existing neuroprotective therapies. Therefore, gaining a better knowledge of the pathogenic mechanisms involved in oxidative stress may provide a hint for identifying treatment targets for ischemic stroke.

Nuclear factor erythroid 2‐related factor 2 (Nrf2) is a master transcription factor that is thought to be the guardian of redox equilibrium and a potential therapeutic target for ischemic stroke.^[^ ^6^ ^]^ Normally, Nrf2 interacts with cytoplasmic Kelch‐like ECH‐associated protein‐1 (Keap1) and is degraded by a Cullin 3 (Cul3)‐based ubiquitination complex.^[^ ^7^ ^]^ In response to stimuli, Nrf2 is released from the complex and translocate to the nucleus, where it forms a heterodimer with genes containing antioxidant response elements (AREs) and induces the production of antioxidant enzyme‐encoding genes, including NAD(P)H quinone oxidoreductase 1 (NQO1), heme oxygenase‐1 (HO‐1) and glutamate cysteine ligase (GCL).^[^ ^8^ ^]^ The Nrf2 signaling pathway not only contributes to cellular protection against oxidative stress but also inhibits neuroinflammation.^[^ ^9^ ^]^ Multiple studies have already revealed the importance of Nrf2 in modulating microglial activation in response to neuroinflammation and revealing that Nrf2 is a promising target for therapy in ischemic stroke.^[^ ^10^ ^]^ An extensive investigation found that Nrf2 activity and abundance are strictly controlled by post‐translational modifications (PTMs), including phosphorylation,^[^ ^11^ ^]^ acetylation^[^ ^12^ ^]^ and ubiquitination.^[^ ^13^ ^]^ For example, the phosphorylation of Nrf2 at Y568 controls its nuclear export, while the phosphorylation of Nrf2 at S374/S408/S433 promotes its βTrCP2‐mediated degradation.^[^ ^11^, ^14^ ^]^ SUMOylation is one of the most important PTMs and is known to regulate stability, translocation, activity, and protein‒protein interactions of the substrates.^[^ ^15^ ^]^ Besides the phosphorylation, Smita et al. revealed that Nrf2 could also be modified by SUMOylation, which plays an important role in its transcriptional activity.^[^ ^16^ ^]^ However, the key SUMO modification protein and enzyme that regulate the SUMOylation of Nrf2 and its role in ischemic stroke remain poorly understood.

SENP6 belongs to the sentrin/SUMO‐specific protease (SENP) family, which has six members (SENP1‐3, 5–7) and deconjugates SUMOs from substrate proteins during the SUMOylation process.^[^ ^17^ ^]^ Multiple studies have demonstrated that SENP6 plays crucial roles in modulating cellular protein function, expression, activity, localization, and stability by selectively depolymerizing SUMO chains from the target protein.^[^ ^18^ ^]^ SENP6 plays a key function in the regulation of neuroinflammation and neuronal apoptosis. For instance, SENP6‐induced deSUMOylation of NF‐kappa‐B essential modulator (NEMO) NEMO blocks the activation of the NF‐κB signaling pathway, subsequently suppressing neuroinflammation.^[^ ^19^ ^]^ Furthermore, our previous research revealed that SENP6 increased neuronal damage by modulating the deSUMOylation of annexin‐A1 (ANXA1) following ischemic stroke in neurons.^[^ ^20^ ^]^ Additionally, we demonstrated that SENP6 caused microglial polarization and neuroinflammation following cerebral I/R injury by de‐SUMOylating ANXA1 in microglia.^[^ ^21^ ^]^ However, little is known about the effects of SENP6 on oxidative stress outcomes after ischemic stroke.

In the present study, we attempted to investigate whether SENP6 was crucial in oxidative stress after ischemic stroke and study its underlying mechanism. We demonstrated that Nrf2 was a previously unknown physiological substrate of SENP6 in neurons. Moreover, SENP6 interacted with Nrf2 and mediated the deSUMOylation of Nrf2 at lysine 533 residue, which led to an increase in its ubiquitination modification and subsequent degradation of Nrf2 by the ubiquitin‒proteasome system, resulting in the negative regulation of the Nrf2‐dependent antioxidant response. Additionally, the upregulation of Nrf2 SUMOylation via SENP6 knockdown protected against oxidative stress‐trigged neuronal apoptosis after ischemic stroke. Importantly, a cell membrane‐permeable peptide that preserve Nrf2 SUMOylation by blocking the direct interaction of SENP6 and Nrf2 shows comparable neuroprotective effects, as evidenced by reduced oxidative stress and brain neuronal loss, mitigated neurological deficit scores, and improved long‐term neurological function in an animal model of stroke, which laying the groundwork for the development of potential therapeutic strategies for ischemic stroke.

## 2 Results

### 2.1 SENP6 Mediated Nrf2 deSUMOylation Following Cerebral I/R Injury

**Figure 1:**
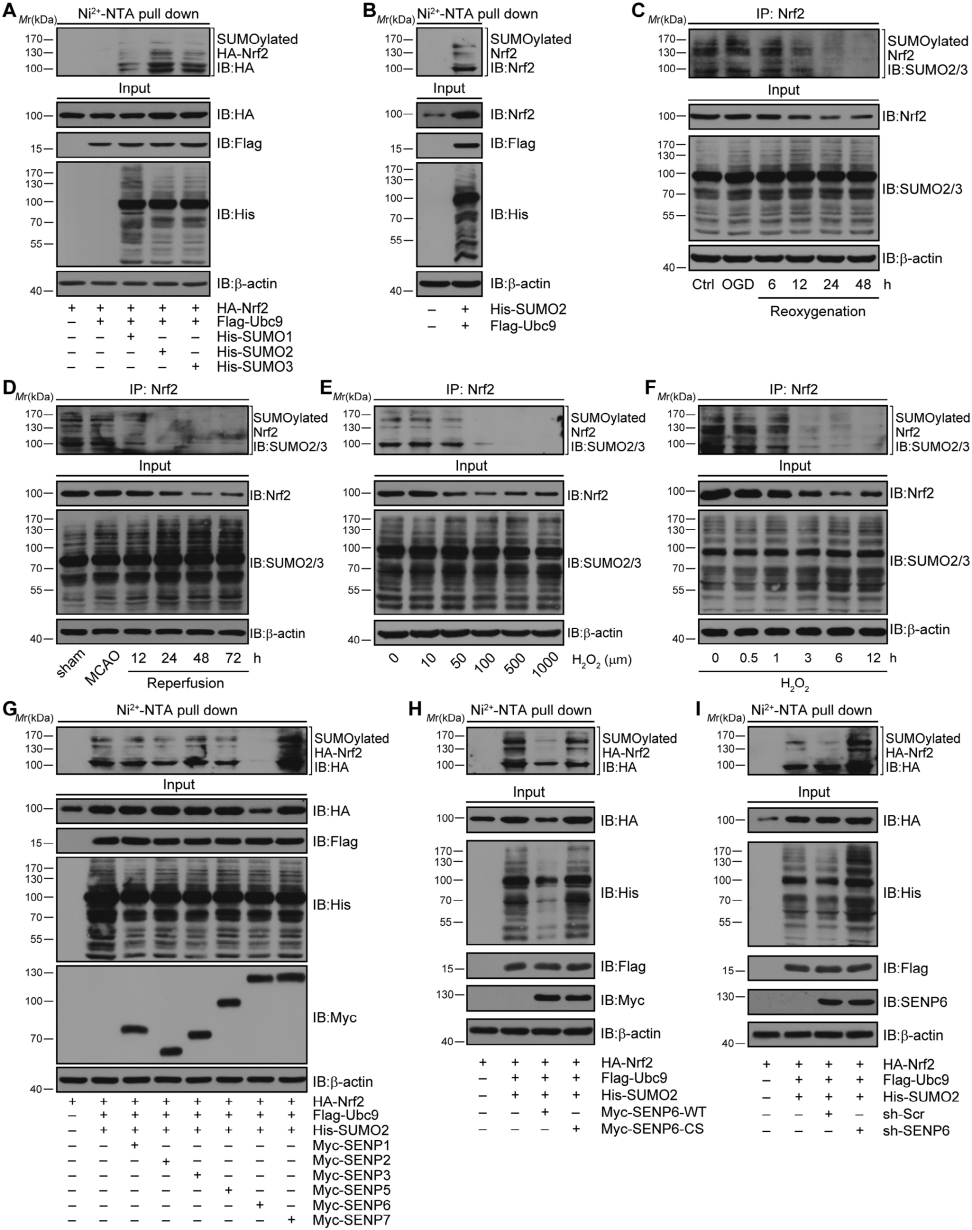
Nrf2 is modified by SUMOylation, and SENP6 mediates the deSUMOylation of Nrf2. A) HA‐Nrf2, His‐SUMO1/2/3 and Flag‐Ubc9 were cotransfected into HEK293T cells for 48 h. Ni^2+^‐NTA pull‐down assays were conducted to determine the SUMOylation of HA‐Nrf2. B) His‐SUMO2 and Flag‐Ubc9 were cotransfected into HEK293T cells for 48 h. Ni^2+^‐NTA pull‐down assays were used to examine the SUMOylation of endogenous Nrf2. C) SUMO2/3 conjugated covalently to ANXA1. Primary neurons were subjected to OGD for 1 h and reperfusion for 6, 12, 24, and 48 h. Cell lysates were used for IP with anti‐Nrf2 antibody, followed by western blot analysis with anti‐SUMO2/3 antibody to detect the SUMOylated band. D) Mouse brain homogenates were extracted at the indicated time points following cerebral ischemia and reperfusion injury, and the lysates were used for IP with anti‐Nrf2 antibody, followed by western blot analysis with anti‐SUMO2/3 antibody to detect the SUMOylated band. E,F) Primary cultured neurons were stimulated with increasing concentrations of H_2_O_2_ for 1 h (E) or H_2_O_2_ (50 mM) for the indicated times (F). Cell lysates were used for IP with anti‐Nrf2 antibody, followed by western blot analysis with anti‐SUMO2/3 antibody to detect the SUMOylated band. G) HEK293T cells were transfected with the indicated plasmids and different Myc‐SENPs. Ni^2+^‐NTA assays were applied to detect HA‐Nrf2 SUMOylation. H) HEK293T cells were cotransfected with HA‐Nrf2, Flag‐Ubc9 and His‐SUMO2 together with Myc‐SENP6‐WT or Myc‐SENP6‐C1030S. Ni^2+^‐NTA agarose was applied to detect HA‐Nrf2 SUMOylation. I) HEK293T cells were treated with SENP6 knockdown by specific shRNA and cotransfected with the indicated plasmids. Ni^2+^‐NTA agarose was applied to detect HA‐Nrf2 SUMOylation. Data represent at least three independent experiments. *M_r_*, relative molecular mass.

First, we determined whether Nrf2 might be modulated by SUMOylation following ischemic stroke. Consistent with previous studies,^[^ ^16^, ^22^ ^]^ Ni^2+^‐NTA (nickel–nitrilotriacetic acid) pull‐down assays demonstrated that Nrf2 was modified mainly by SUMO2 and SUMO3 but weakly by SUMO1 (**Figure** **1**A). Additionally, His‐SUMO2 was transduced into HEK293T cells, and the pull‐down assay demonstrated that SUMO2 could also modify endogenous Nrf2 (Figure 1B). Therefore, in subsequent research, we concentrated on the Nrf2 SUMO2/3 modification. Subsequently, a time course investigation demonstrated that the SUMOylation level of Nrf2 in neurons was steadily reduced following oxygen‐glucose deprivation and reperfusion (OGD/R) (Figure 1C). In concordance with the in vitro results, coimmunoprecipitation (Co‐IP) experiments also confirmed that the SUMOylation of Nrf2 gradually downregulated in mice after the onset of ischemic stroke (Figure 1D). Since Nrf2 has been demonstrated to regulate the oxidative stress response, we examined the SUMOylation of Nrf2 in neurons exposed to H_2_O_2_, the direct inducer of oxidative stress. Co‐IP assays revealed that the SUMOylation of Nrf2 was greatly downregulated in a dose‐ and time‐dependent manner (Figure 1E,F). Next, our investigation aimed to elucidate the specific SENP enzyme responsible for the deSUMOylation of Nrf2. HEK293T cells were transiently transduced with a panel of Myc‐SENPs together with HA‐Nrf2. The results demonstrated that SENP6, but not the other members of SENP family, resulted in a markedly reduction in the SUMOylation of Nrf2 (Figure 1G). Next, we transfected a plasmid containing either the wild‐type (WT) or a catalytic mutant form of SENP6 into cells. In contrast to WT SENP6, SENP6‐C1030S, which contains a mutation at position 1030 where cysteine is replaced by serine, resulted in the loss of its enzyme activity to deconjugate SUMO from Nrf2 (Figure 1H). Additionally, the results revealed that SUMOylation of Nrf2 was enhanced when SENP6 was downregulated by a specific short hairpin RNA (shRNA) (Figure 1I). Collectively, these results provide strong evidence that Nrf2 can be modified by SUMOylation and SENP6 effectively removes SUMO2/3 chains from Nrf2 following ischemic stroke.

### 2.2 SENP6 Interacts with and deSUMOylates Nrf2 at Lysine Residue 533 After OGD/R

**Figure 2:**
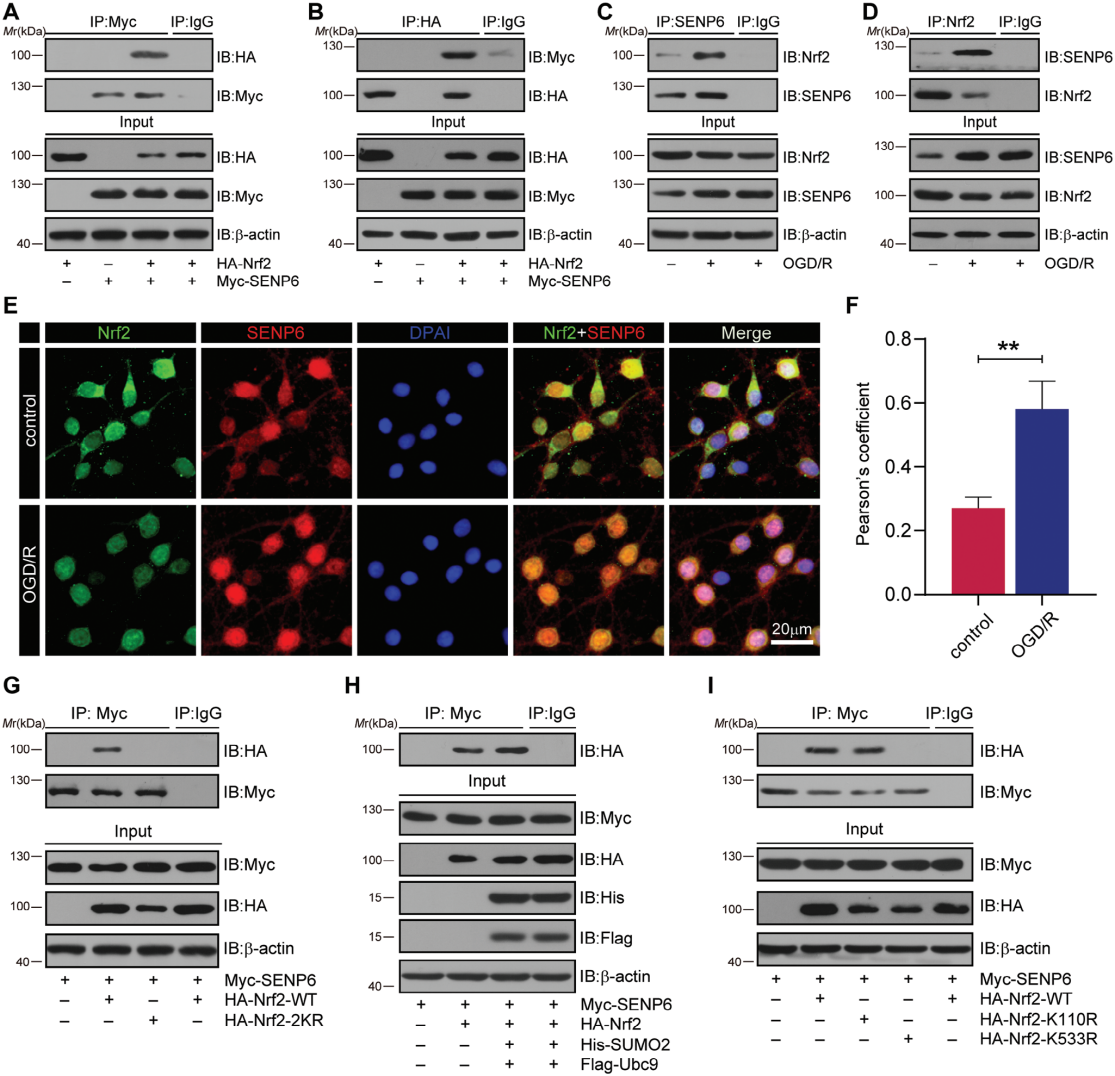
SENP6 interacts with and deSUMOylates Nrf2 at lysine 533 after OGD/R. A,B) HEK293T cells were cotransfected with HA‐Nrf2 and Myc‐SENP6 for 48 h. Co‐IP was applied to examine the exogenous binding of SENP6 and Nrf2. C,D) Primary neurons were challenged with OGD for 1 h and reperfusion for 24 h. Co‐IP was applied to examine the endogenous binding of SENP6 and Nrf2, and immunoblot assays was conducted using anti‐Nrf2, anti‐SENP6 and anti‐*β*‐actin antibodies. E) Primary neurons were subjected to OGD/R, and immunofluorescence analysis was applied to determine the colocalization of Nrf2 and SENP6. Scale bar = 20 µm. F) Quantitative analysis of the colocalization was performed by Pearson's coefficient measurement by ImageJ software. G) HEK293T cells were transfected with Myc‐SENP6 together with HA‐Nrf2‐WT or HA‐Nrf2‐2KR for 48 h. Co‐IP was used to determine the binding between SENP6 and HA‐Nrf2‐WT or HA‐Nrf2‐2KR. H) HEK293T cells were cotransfected with Myc‐SENP6 and HA‐Nrf2 along with His‐SUMO2 and Flag‐Ubc9 for 48 h. Co‐IP was applied to examine the binding of SENP6 and Nrf2. I) HEK293T cells were transfected with Myc‐SENP6 along with HA‐Nrf2‐WT, HA‐Nrf2‐K110R or HA‐Nrf2‐K533R for 48 h. Co‐IP was applied to examine the interaction of SENP6 with HA‐Nrf2‐WT/K110R/K533R. Data are shown as the mean ± SD and quantified by the unpaired two‐tailed Student's t test. n = 3; **p < 0.01.

To confirm whether Nrf2 is a substrate of SENP6, we first detected the expression of SENP6 and Nrf2 in primary cultured neurons after OGD/R. The immunoblot assays revealed that the protein levels of SENP6 increased at 12 h and peaked at 24 h after OGD, whereas the protein levels of Nrf2 showed the opposite trend (Figure S1, Supporting Information). Next, we determine the interaction between Nrf2 and SENP6. As presented in **Figure** **2**A, the binding of ectopically expressed HA‐Nrf2 to Myc‐SENP6 was detected by Co‐IP in HEK293T cells. This finding was also validated by reverse Co‐IP tests (Figure 2B). Moreover, the interaction between endogenous Nrf2 and SENP6 was validated by Co‐IP in primary neurons. The findings showed that OGD/R notably enhanced the binding of Nrf2 to SENP6 (Figure 2C,D). In addition, we examined the subcellular location of the interaction in primary cultured neurons, which suggested that OGD/R increased SENP6‐Nrf2 interactions and that this interaction occurred both in the cytoplasm and nucleus (Figure S2A, Supporting Information). Subsequently, double immunofluorescence analysis demonstrated a notable increase in the colocalization of SENP6 with Nrf2 in neurons after OGD/R damage (Figure 2E,F). We then aimed to determine the deSUMOylation site(s) of Nrf2. Previous research has reported that lysine 110 and 533 (K110/533) are the major SUMO modification sites of human Nrf2.^[^ ^16^ ^]^ Then, these two lysine residues, were mutated to arginine for the simulation of the deSUMOlytion state, including K110R, K533R, and K110/533R (2KR, lysine‐to arginine at residues 110 and 533). First, HEK293T cells were transduced with Myc‐SENP6 along with either HA‐Nrf2‐WT or Nrf2‐2KR. The results indicated that SENP6 could interact with Nrf2‐WT rather than with Nrf2‐2KR (Figure 2G). Then, HEK293T cells were cotransfected with Myc‐SENP6, HA‐Nrf2‐WT and His‐SUMO2 together with Flag‐Ubc9, the only E2‐conjugating enzyme that is important for SUMOylation.^[^ ^23^ ^]^ Co‐IP assays demonstrated that the SUMOylation of Nrf2 increased the interaction of Nrf2‐WT with SENP6 (Figure 2H). Additionally, Myc‐SENP6 together with HA‐Nrf2‐WT, Nrf2‐K110R or Nrf2‐K533R was transfected into cells. Co‐IP assays indicated that SENP6 interacted with Nrf2‐WT and Nrf2‐K110R but not with Nrf2‐K533R (Figure 2I). Furthermore, Ni^2+^‐NTA pull‐down assays revealed that the K533R mutation significantly reduced SUMOylation levels of Nrf2 (Figure S2B, Supporting Information). Since K533 is located within the Neh1 domain of the Nrf2, we next verified whether Nrf2 binds to the SENP6 through the Neh1 domain. The full‐length Nrf2 and the ΔNeh1 mutant constructs with SENP6 were co‐transfected into HEK293 cells, and Co‐IP assays confirmed that the Neh1 domain of Nrf2 was critical for the interaction with SENP6 (Figure S2C, Supporting Information). Collectively, these data revealed that SENP6 deSUMOylates Nrf2 at the C‐terminal residue K533 via a direct interaction after ischemic stroke.

### 2.3 SENP6‐Induced deSUMOylation Facilitates Ubiquitination‐Dependent Degradation of Nrf2

**Figure 3:**
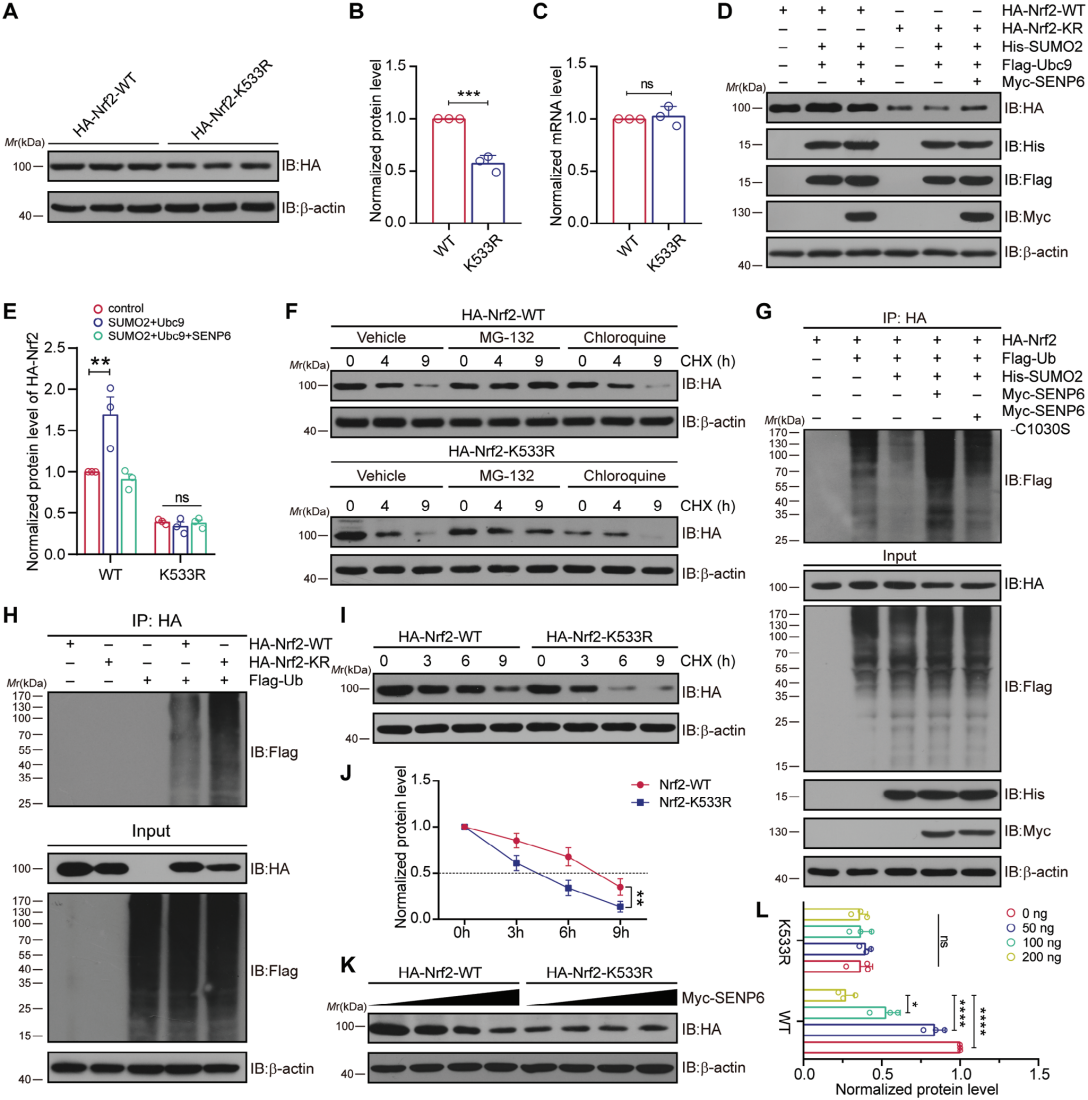
SENP6‐mediated deSUMOylation of Nrf2 promotes its ubiquitin proteasome‐dependent degradation. A) HA‐Nrf2‐WT or HA‐Nrf2‐K533R were transfected into HEK293T cells. Immunoblot analysis was applied to determine the expression of ectopically expressed Nrf2. B) Quantitative analysis of ectopically expressed Nrf2 levels in (A). C) qPCR was conducted to detected the mRNA levels of *Nfe2l2*, which encodes the Nrf2 protein. D) HEK293T cells were transfected with HA‐Nrf2‐WT or HA‐Nrf2‐K533R together with His‐SUMO2, Flag‐Ubc9 or Myc‐SENP6 for 48 h. Immunoblot analysis was applied to examine the levels of ectopically expressed Nrf2. E) Quantitative analysis of ectopically expressed Nrf2 protein expression in (D). F) HEK293T cells were transduced with HA‐Nrf2‐WT or HA‐Nrf2‐K533R for 48 h and then treated with CHX and MG‐132 or chloroquine for the indicated times. Immunoblot assays was applied to detected the levels of ectopically expressed Nrf2. G) HEK293T cells were transfected with HA‐Nrf2, His‐SUMO2 and Flag‐Ub together with Myc‐SENP6‐WT or Myc‐SENP6‐C1030S for 48 h. Co‐IP was applied to determine the polyubiquitination level of Nrf2. H) HEK293T cells were cotransfected with Flag‐Ub together with HA‐Nrf2‐WT or HA‐Nrf2‐K533R for 48 h. Co‐IP was applied to determine the polyubiquitination level of Nrf2. I) HEK293T cells were transduced with HA‐Nrf2‐WT or HA‐Nrf2‐K533R for 48 h and subjected to CHX for different times. Immunoblot analysis was applied to determine the half‐life of ectopically expressed Nrf2. J) Quantitative analysis of Nrf2 levels in (I). K) HEK293T cells were transfected with HA‐Nrf2‐WT or HA‐Nrf2‐K533R along with increasing amounts of Myc‐SENP6 for 48 h. Immunoblot analysis was performed to determine the levels of ectopically expressed Nrf2. L) Quantitative analysis of HA‐Nrf2 levels in (K). Data are shown as the mean ± SD and quantified by unpaired two‐tailed Student's t test (B and C) or two‐way ANOVA followed by Tukey's post hoc test (E, J and L). n = 3; ns: no significant difference; *p < 0.05, **p < 0.01, ***p < 0.001 and ****p < 0.0001.

Since SUMOylation modulates the functions of targeted proteins, including protein stability,^[^ ^15^ ^]^ it was fascinating to investigate whether SENP6‐induced deSUMOylation of Nrf2 modulates its stability. First, HA‐Nrf2‐WT and Nrf2‐K533R were transduced into HEK293T cells, immunoblot assays revealed that the protein levels of Nrf2‐K533R obviously decreased as compared to Nrf2‐WT (**Figure** **3**A,B). However, the quantitative real‐time polymerase chain reaction (qPCR) data revealed no difference in the levels of *Nfe2l2* mRNA, which encodes the Nrf2 protein (Figure 3C). These findings suggested that the decreased Nrf2 abundance occurred at the protein level. Then, immunoblot showed that the levels of Nrf2 notably increased when Nrf2‐WT was cotransfected with His‐SUMO2 and Flag‐Ubc9, but decreased when cotransfected with Myc‐SENP6. In addition, no significant difference in the expression level of Nrf2‐K533R was observed when Nrf2‐K533R was cotransfected with either His‐SUMO2 or Myc‐SENP6 (Figure 3D,E). Overall, these data indicated that SENP6‐mediated deSUMOylation of Nrf2 promoted its degradation.

Next, the precise molecular mechanism behind the degradation of deSUMOylated Nrf2 were studied. The ubiquitin‒proteasome system and the autophagy‒lysosome pathway are two major mechanisms that eukaryotic cells employ for protein degradation.^[^ ^24^ ^]^ Pharmacologic methods were utilized to determine which pathway is responsible for Nrf2 clearance. Immunoblot assays indicated that deSUMOylation‐induced degradation of Nrf2‐WT and Nrf2‐K533R was abolished by the proteasome inhibitor MG‐132, but not by the autophagy inhibitor chloroquine (CQ) (Figure 3F). This finding suggests that the clearance of Nrf2 occurred mostly via the ubiquitin‒proteasome system, rather than the autophagy‒lysosome pathway.

SUMOylation and ubiquitination can act either synergistically or antagonistically.^[^ ^25^ ^]^ To investigate the impact of SUMOylation on Nrf2 ubiquitination, HEK293T cells were cotransfected with HA‐Nrf2, Flag‐ubiquitin (Ub) together with His‐SUMO2, Myc‐SENP6 or Myc‐SENP6‐C1030S. Co‐IP assays revealed that SUMOylated Nrf2 obviously inhibited its polyubiquitination. Moreover, SENP6‐WT overexpression significantly enhanced Nrf2 polyubiquitination, while SENP6‐C1030S had little effects (Figure 3G). In addition, Co‐IP assays showed that the polyubiquitination of Nrf2‐K533R significantly increased compared to that of HA‐Nrf2‐WT (Figure 3H). Furthermore, the inhibition of de novo protein synthesis by cycloheximide (CHX) was conducted to determine the time course of deSUMOylation–trigged Nrf2 degradation. As expected, the turnover rate for HA‐Nrf2‐K533R was obviously accelerated (Figure 3I,J). This finding indicates that the degradation of SUMOylated Nrf2 is slowed down. Finally, we transfected HEK293T cells with increasing amounts of Myc‐SENP6 (0, 50, 100, and 200 ng) together with HA‐Nrf2‐WT or HA‐Nrf2‐K533R. The immunoblot tests revealed that increased dosages of Myc‐SENP6 significantly reduced the levels of HA‐Nrf2‐WT protein but had no impact on the levels of HA‐Nrf2‐K533R protein (Figure 3K,L). Collectively, these data show that the deSUMOylation of Nrf2 by SENP6 plays a critical role in controlling its degradation through the ubiquitin‒proteasome system.

### 2.4 SENP6 Negatively Regulates the Activation of the Nrf2‐ARE Signal Pathway Following OGD/R

**Figure 4:**
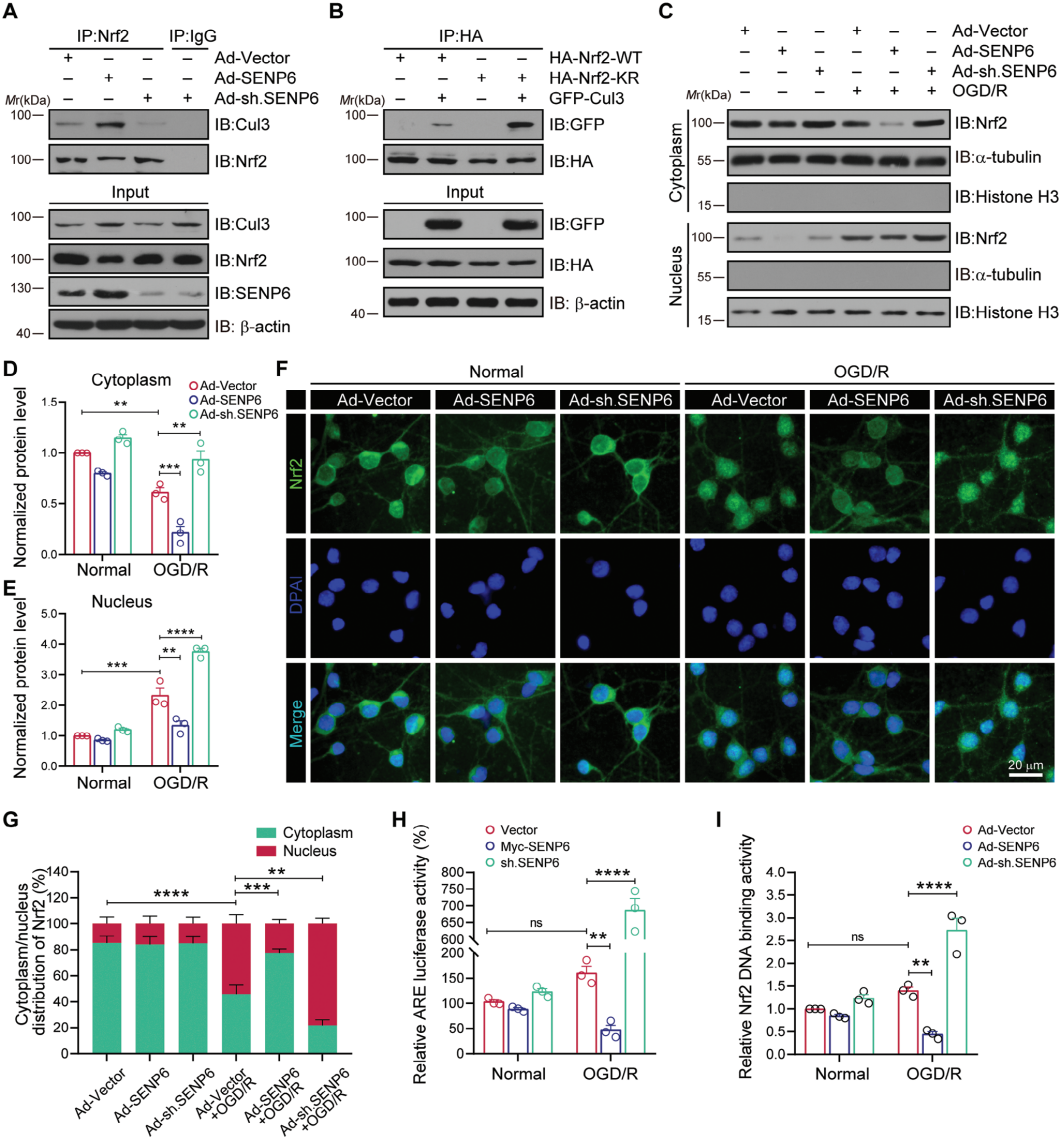
SENP6 blocked activation of the Nrf2‐ARE signal pathway after OGD/R. A) Recombinant adenoviruses expressing the SENP6 coding sequence (Ad‐SENP6), the SENP6 shRNA sequence (Ad‐sh. SENP6) or the empty vector (Ad‐vector) were infected into primary cultured neurons for 48 h. Co‐IP was performed to detected the endogenous binding between Cul3 and Nrf2. B) HEK293T cells were cotransfected with GFP‐Cul3 together with HA‐Nrf2‐WT or HA‐Nrf2‐K533R for 48 h. Co‐IP was applied to determine the interaction between GFP‐Cul3 and HA‐Nrf2‐WT or HA‐Nrf2‐K533R. C) Primary neurons were transfected with recombinant adenoviruses encompassing SENP6 or shRNA for 48 h and subjected to OGD/R. Immunoblot assays was applied to examine the levels of endogenous Nrf2 in cytoplasmic or nuclear fragments. D,E) Quantitative analysis of Nrf2 levels in (C). F) Immunofluorescence analysis was applied to determine the subcellular location of Nrf2. Scale bar = 20 µm. G) Statistical analysis of the nuclear or cytoplasmic Nrf2 levels in (F). H) HEK293T cells were transiently transfected with ARE‐luciferase or control vector together with Myc‐SENP6 or SENP6 shRNA for 48 h and subjected to OGD/R. Luciferase activity was examined by an analyzer fluorescence assay. I) The DNA binding activity of Nrf2 in primary cultured neurons was determined by an ELISA‐based (Trans‐AM) method. Data are shown as the mean ± SD and quantified by two‐way ANOVA followed by Tukey's post hoc test. n = 3; ns: no significant difference; *p < 0.05, **p < 0.01, ***p < 0.001 and ****p < 0.0001.

After confirming the deSUMOylation of Nrf2 has an effect on its protein stability, we then attempted to assess the potential role of SENP6 as a modulator of Nrf2 activity. Cul3, as a scaffolding protein, constitutes an important member of the E3 ubiquitin‐protein ligase complex, which has substrate specificity and negatively affects the Nrf2 signaling pathway. In addition, Keap1 serves as a bridge between Nrf2 and the Cul3‐based E3‐ligase ubiquitination complex.^[^ ^26^ ^]^ First, we conducted Co‐IP experiments to examine the binding of Cul3 and Keap1 with Nrf2. For this purpose, primary neurons were infected with adenoviruses expressing SENP6‐WT or SENP6 shRNA. The Co‐IP data demonstrated that the interaction of Cul3 and Nrf2 increased after SENP6 upregulation but decreased after SENP6 knockdown (**Figure** **4**A). Moreover, SENP6 had the same effect on Keap1‐Nrf2 binding (Figure S3, Supporting Information). Next, HEK293T cells were cotransfected with GFP‐Cul3 and HA‐Nrf2‐WT or HA‐Nrf2‐K533R. Co‐IP assays indicated that the interaction of HA‐Nrf2‐K533R with Cul3 was significantly enhanced compared to that of HA‐Nrf2‐WT (Figure 4B). In addition, in view of the important role of the subcellular localization of Nrf2 in oxidative stress, we next explored whether SENP6 modulates the nuclear translocation of Nrf2. As displayed in Figure 4C–E, immunoblots indicated that OGD/R decreased the amount of Nrf2 in the cytoplasmic fractions and enhanced the entry of Nrf2 into the nuclear fractions. SENP6 upregulation reduced the nuclear levels of Nrf2. However, SENP6 downregulation significantly increased the nuclear levels of Nrf2. These data were also verified by immunofluorescence assay (Figure 4F,G). Additionally, we conducted further experiments to determine whether SENP6 regulated Nrf2 transcriptional activity. The luciferase reporter assay demonstrated that the upregulation of SENP6 led to a downregulation in ARE‐dependent luciferase activity. Conversely, the downregulation of SENP6 resulted in a large increase in ARE luciferase activity (Figure 4H). Finally, we detected a promotion of Nrf2 DNA‐binding activity in neurons as a result of knockdown of SENP6 (Figure 4I). The results indicate that SENP6 blocks the nuclear translocation of Nrf2 and suppress its transcriptional activity following OGD/R.

Previous study demonstrated that p300/CBP‐dependent acetylation are critical for the DNA binding of Nrf2 during the antioxidant response.^[^ ^27^ ^]^ To understand the mechanisms underlying SENP6 knockdown‐induced Nrf2 DNA‐binding, we further explored the relationship between K533 SUMOylation and p300/CBP‐mediated acetylation. Adenoviruses encoding SENP6‐WT and SENP6 shRNA were used to infect primary cultured neurons. The immunoblot results revealed that SENP6 overexpression caused a substantial decrease in Nrf2 acetylation, while SENP6 knockdown greatly induced the acetylation of Nrf2 (Figure S4A,B, Supporting Information). In addition, we found that SENP6 overexpression markedly decreased the interaction of Nrf2 with p300. However, SENP6 knockdown exhibited the opposite effect (Figure S4C,D, Supporting Information). Taken together, the above data indicated that SENP6 could decrease Nrf2 acetylation.

### 2.5 SENP6 Inhibits Nrf2‐Dependent Antioxidant Response after OGD/R

**Figure 5:**
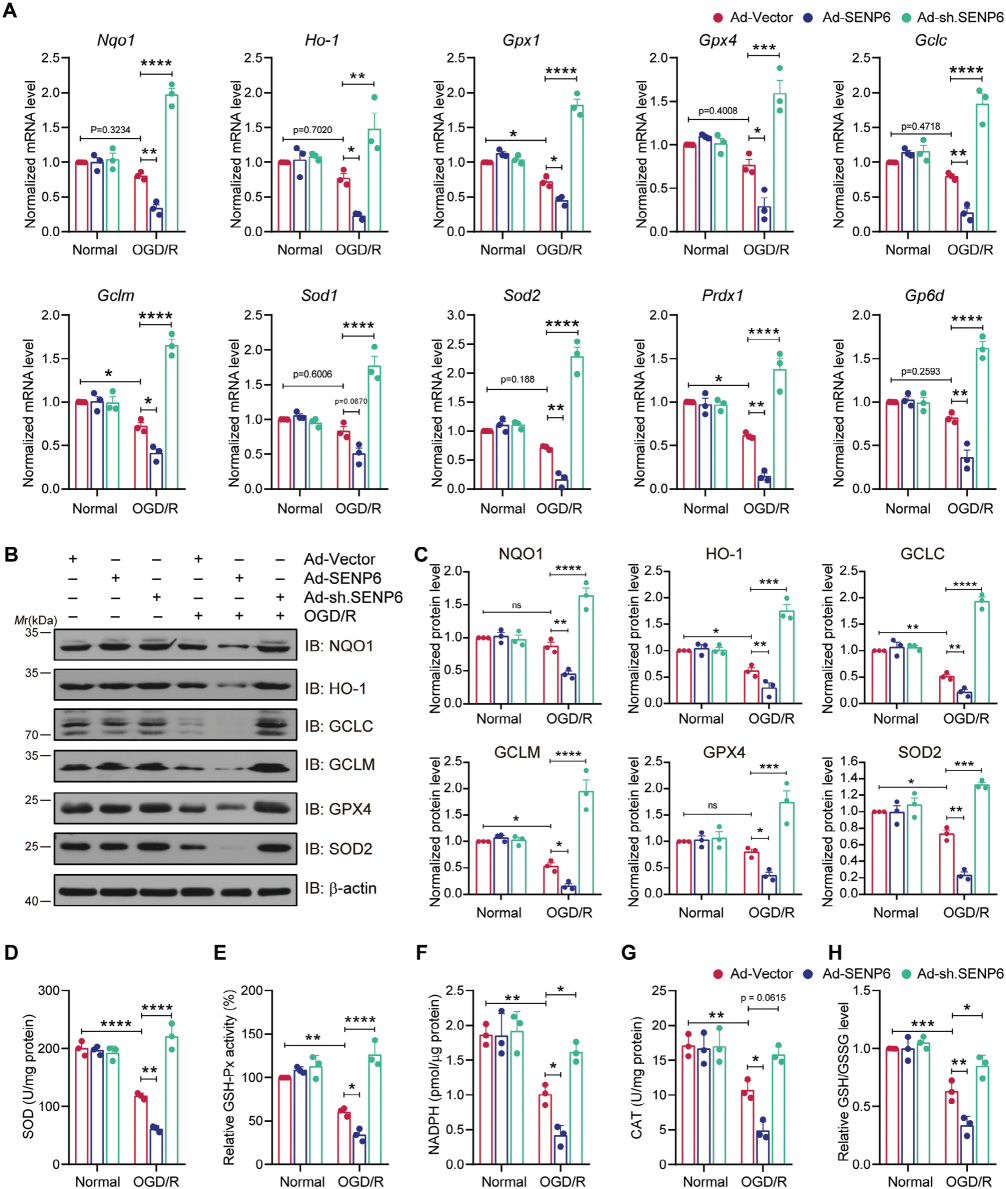
SENP6 decreased Nrf2‐targeted antioxidant enzyme expression and activation after ischemic stroke. Primary neurons were transfected with recombinant adenoviruses encoding vector, SENP6, or shRNA for 48 h and then challenged with OGD/R. A) qPCR was used to examine the mRNA levels of *Nqo‐1*, *Ho‐1*, *Gpx1*, *Gpx4*, *Gclc*, *Gclm*, *Sod1*, *Sod2*, *Prdx1* and *Gp6d*. B) Immunoblot analysis was applied to detected the protein expression of NQO1, HO‐1, GCLC, GCLM, GPX4 and SOD2. C) Statistical analysis of the antioxidant enzyme protein levels in (B). D‐H) The results showing the effects of SENP6 overexpression or knockdown on the activity of SOD, GSH‐Px, NADPH, CAT and GSH/GSSG levels. Data are shown as the mean ± SD and quantified by two‐way ANOVA followed by Tukey's post hoc test. n = 3; ns: no significant difference; *p < 0.05, **p < 0.01, ***p < 0.001 and ****p < 0.0001.

Nrf2 targets several genes with AREs in their promoters.^[^ ^28^ ^]^ We then examine the ability of SENP6 to regulate Nrf2‐targeted antioxidant response. Neuronal cells were transfected with adenoviruses expressing SENP6‐WT or SENP6 shRNA for 48 h and then subject to OGD/R. As presented in **Figure** **5**A, qPCR analysis showed that SENP6 upregulation reduced the mRNA levels of *Nqo‐1*, *Ho‐1*, *Gpx1*, *Gpx4*, *Gclc*, *Gclm*, *Sod1*, *Sod2*, *Prdx1* and *Gp6d* after OGD/R. However, this phenomenon was significantly reversed by SENP6 knockdown. Moreover, the protein levels of the Nrf2‐targeted antioxidant gene were also examined. Consistent with the qPCR results, immunoblots indicated that the protein levels of NQO‐1, HO‐1, GCLC, GCLM, GPX4 and SOD2 was significantly decreased after SENP6 upregulation. However, this phenomenon was reversed by SENP6 knockdown, showing that levels of the above antioxidant enzyme proteins obviously increased after OGD/R (Figure 5B,C). Furthermore, we examined the activities of superoxide dismutase (SOD), glutathione peroxidase (GSH‐Px), nicotinamide adenine dinucleotide phosphate (NADPH), catalase (CAT) and ROS. The results revealed that SENP6 upregulation decreased the activities of the antioxidant enzymes, but SENP6 downregulation significantly elevated the activities of the indicated enzymes (Figure 5D,G). Finally, we compared the overall redox state. As shown in Figure 5H, SENP6 upregulation further decreased the glutathione (GSH)/oxidized glutathione (GSSG) ratio compared with OGD/R, but SENP6 downregulation significantly reversed this phenomenon. Collectively, these findings indicate that SENP6 negatively regulates the expression of Nrf2‐targeted genes and inhibits the antioxidant response in neurons under OGD/R conditions.

### 2.6 SENP6 Induces Oxidative Stress Injury and Increases Neuronal Damage Following OGD/R

**Figure 6:**
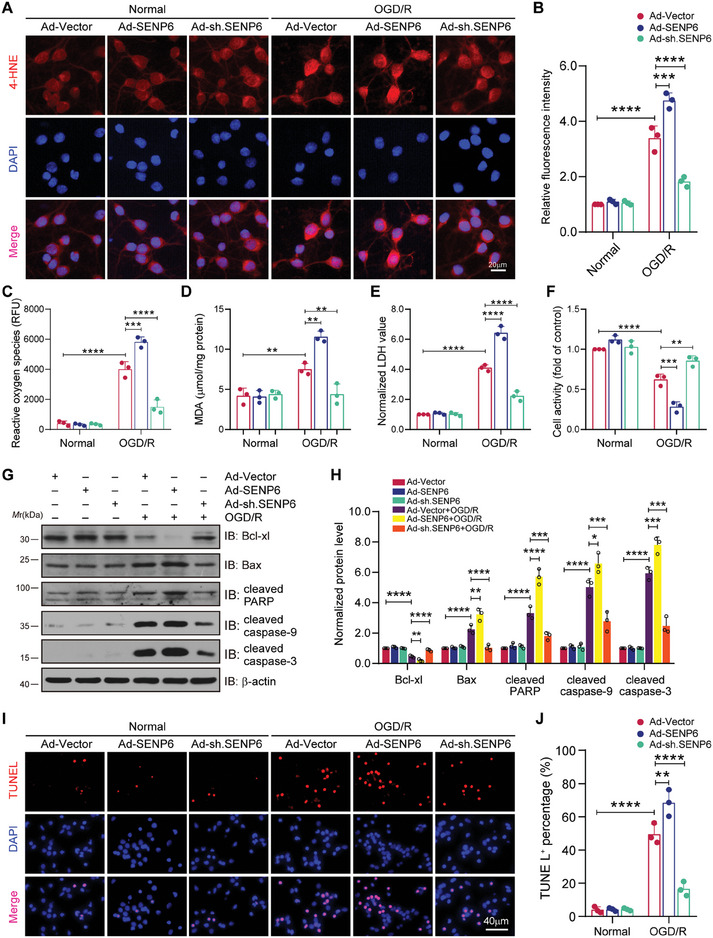
SENP6 promotes oxidative stress damage and induces neurotoxicity after ischemic stroke. Primary neurons were transfected with recombinant adenoviruses encoding vector, SENP6, or shRNA for 48 h and then challenged with OGD/R. A) Immunofluorescence analysis was applied to determine the levels of 4‐HNE as an indicator of lipid peroxidation. Scale bar = 20 µm. B) Statistical analysis of the fluorescence intensities of 4‐HNE. C) Quantitative analysis of ROS measurements. D) Results showing the level of MDA. E) Colorimetry was applied to determine the LDH released from neurons. F) Neuronal viability was examined by CCK‐8 assay. G) Immunoblot assays was conducted to detected the protein levels of Bcl‐xl, Bax, cleaved PARP, cleaved caspase‐9 and cleaved caspase‐3. H) Quantitative analysis of the indicated protein levels in (G). I) TUNEL staining was performed to determine the number of apoptotic cells. Scale bar = 40 µm. J) Statistical analysis of TUNEL‐positive cells in (I). Data are expressed as the mean ± SD and quantified by two‐way ANOVA followed by Tukey's post hoc test. n = 3; **p < 0.01, ***p < 0.001 and ****p < 0.0001.

We then explored the impact of SENP6 on oxidative stress‐induced neuronal damage. For this purpose, neuronal cells were transfected with adenoviruses expressing SENP6‐WT or SENP6 shRNA for 48 h and then challenged with OGD/R. First, immunofluorescence analysis revealed that SENP6 upregulation further enhanced the fluorescence intensity of oxidative stress marker 4‐hydroxynonenal (4‐HNE) induced by OGD/R, but SENP6 knockdown obviously reversed this phenomenon (**Figure** **6**A,B). Second, we measured a broad range of ROS in neurons, demonstrating that a significant increase in ROS was detected after SENP6 overexpression but a reduction after SENP6 knockdown (Figure 6C). In line with these results, SENP6 upregulation further enhanced the level of MDA induced by OGD/R, but SENP6 knockdown obviously reversed this trend (Figure 6D). Moreover, lactate dehydrogenase (LDH) assays showed that SENP6 upregulation further promoted LDH release, whereas SENP6 knockdown significantly alleviated this increase (Figure 6E). Subsequently, neuronal cells viability was detected by a cell counting kit‐8 (CCK‐8) assay, showing that SENP6 upregulation reduced cell viability in neurons after OGD/R, but SENP6 downregulation promoted neuronal viability (Figure 6F). Concurrently, we also employed immunoblot assays to investigate the protein expression of Bcl‐xl, an antiapoptotic molecule, and proapoptotic molecules, such as Bax, cleaved PARP, cleaved caspase‐9, and cleaved caspase‐3. The results indicated that SENP6 knockdown significantly reduced the protein level of proapoptotic molecules but increased the level of antiapoptotic molecules after OGD/R. In contrast, SENP6 overexpression showed the opposite effects (Figure 6G,H). Furthermore, terminal deoxynucleotidyl transferase dUTP nick end labeling (TUNEL) staining showed that SENP6 knockdown significantly alleviated neuronal apoptosis after OGD/R, whereas SENP6 overexpression showed the opposite effects (Figure 6I,J). We next study whether overexpression of Nrf2 could reversed SENP6‐iduced oxidative stress damage and neurotoxicity after ischemic stroke. As shown in Figure S5 (Supporting Information), Nrf2 overexpression significantly reversed SENP6‐induced ROS production, MDA upregulation, LDH release and neuronal apoptosis. Finally, we examined whether the antioxidants *N*‐acetyl‐L‐cysteine (NAC) can block the promotive role of SENP6 on neuron cell death. Primary neurons were infected with recombinant adenoviruses encoding SENP6 for 48 h and then pretreated with NAC for 60 min, after that, the cells were challenged with OGD/R. Immunoblot assays demonstrated that SENP6 significantly increased the protein level of proapoptotic molecules but decreased the level of antiapoptotic molecules after OGD/R, but NAC treatment obviously exhibited the reverse effect (Figure S6A,B, Supporting Information). Additionally, TUNEL staining revealed that SENP6‐induced neuron death was significantly reduced by NAC treatment (Figure S6C,D, Supporting Information). Collectively, these data suggest that SENP6 knockdown decreases oxidative stress injury and subsequently reduces neuronal damage after ischemic stroke.

### 2.7 Tat‐Nrf2 Peptide Administration Attenuates Oxidative Stress After Ischemic Stroke in Mice

Based on the above data, we concluded that blocking the deSUMOylation of Nrf2 induced by SENP6 can protect against neuronal injury. Hence, we hypothesized that inhibiting SENP6‐mediated deSUMOylation of Nrf2 can alleviates ischemic brain injury. According to our previous experience, we synthesized a series of peptides of varying lengths that exhibit overlap with the lysine 525 residue of mouse Nrf2, which is lysine 533 homologous site of human Nrf2. This particular residue is recognized as the binding site for SENP6 (Figure S7A, Supporting Information). Additionally, Co‐IP assays were conducted to examine the efficacy of the indicated peptides. We found that only peptide 3 attenuated the interaction of SENP6 and Nrf2 (Figure S7B, Supporting Information). Thus, we named this peptide Tat‐Nrf2, and we then conducted a dose‒response analysis to examine the effective dose in vitro and in vivo. First, HEK293T cells were cotransfected with Myc‐SENP6, HA‐Nrf2 for 48 h and then treated with Tat‐Nrf2 peptide at single doses of 5, 10, 15, and 20 µM. Co‐IP assays showed that Tat‐Nrf2 at a dose of 15 µM efficiently blocked the interaction of SENP6 and Nrf2 (Figure S7C, Supporting Information). Additionally, neurons were challenged with OGD/R and then treated with Tat‐Nrf2 at single doses of 5, 10, 15, and 20 µM. Co‐IP showed that Tat‐Nrf2 at a dose of 15 µM efficiently preserved the SUMOylation of Nrf2 (Figure S7D, Supporting Information).

To examine the specificity of the effect of Tat‐Nrf2 peptide on SENP6‐Nrf2 interaction, we investigated the effects of Tat‐Nrf2 on the SUMOylation of ANXA1, which is a confirmed substrate of SENP6, and we previously had demonstrated that SENP6 can bind to and mediated the deSUMOylation of ANXA1 after ischemic stroke.^[^ ^20^, ^21^ ^]^ As presented in Figure S8 (Supporting Information), Tat‐Nrf2 had no impact on the binding of SENP6 with ANXA1 and the SUMOylation level of ANXA1 after OGD/R. These data indicated that Tat‐ Nrf2 is a specific inhibitor that can be used to block SENP6‐Nrf2 interaction without affecting the interaction of other substrate proteins of SENP6.

We then investigated whether Tat‐Nrf2 peptide treatment induced ischemic protection in vivo. Frist, fluorescent labels were used to observe the efficient blood‐brain barrier permeability and wide distribution of the Tat‐Nrf2 peptides injected through the tail vein of mice after ischemic stroke (Figure S9, Supporting Information). Then, we administered Tat‐Nrf2 peptide to the mice 6 h after reperfusion at single doses of 5, 10 and 15 mg kg^−1^ for 3 consecutive days via intravenous injections (i.v., Figure S10A, Supporting Information). Co‐IP assays showed that Tat‐Nrf2 at a dose of 10 mg kg^−1^ efficiently decreased the binding of SENP6 to Nrf2 and increased the SUMOylation of Nrf2 (Figure S10B,C, Supporting Information). Next, we administered it to the mice 6 h after reperfusion at a dose of 10 mg kg^−1^ for 3 consecutive days and performed behavioral and histological studies at the indicated time points. Immunoprecipitation revealed that Tat‐Nrf2 treatment obviously inhibited Nrf2 polyubiquitination after ischemic stroke (Figure S11A, Supporting Information). Furthermore, immunoblots showed that Tat‐Nrf2 treatment markedly enhanced the nuclear translocation of Nrf2 after middle cerebral artery occlusion (MCAO) surgery (Figure S11B,C, Supporting Information). Finally, we detected a promotion of Nrf2 DNA‐binding activity in the ischemic penumbra of brain tissue from mice injected with Tat‐Nrf2 peptide (Figure S11D, Supporting Information). Consequently, a dose of 10 mg kg^−1^ was adopted in the remainder of the research.

**Figure 7:**
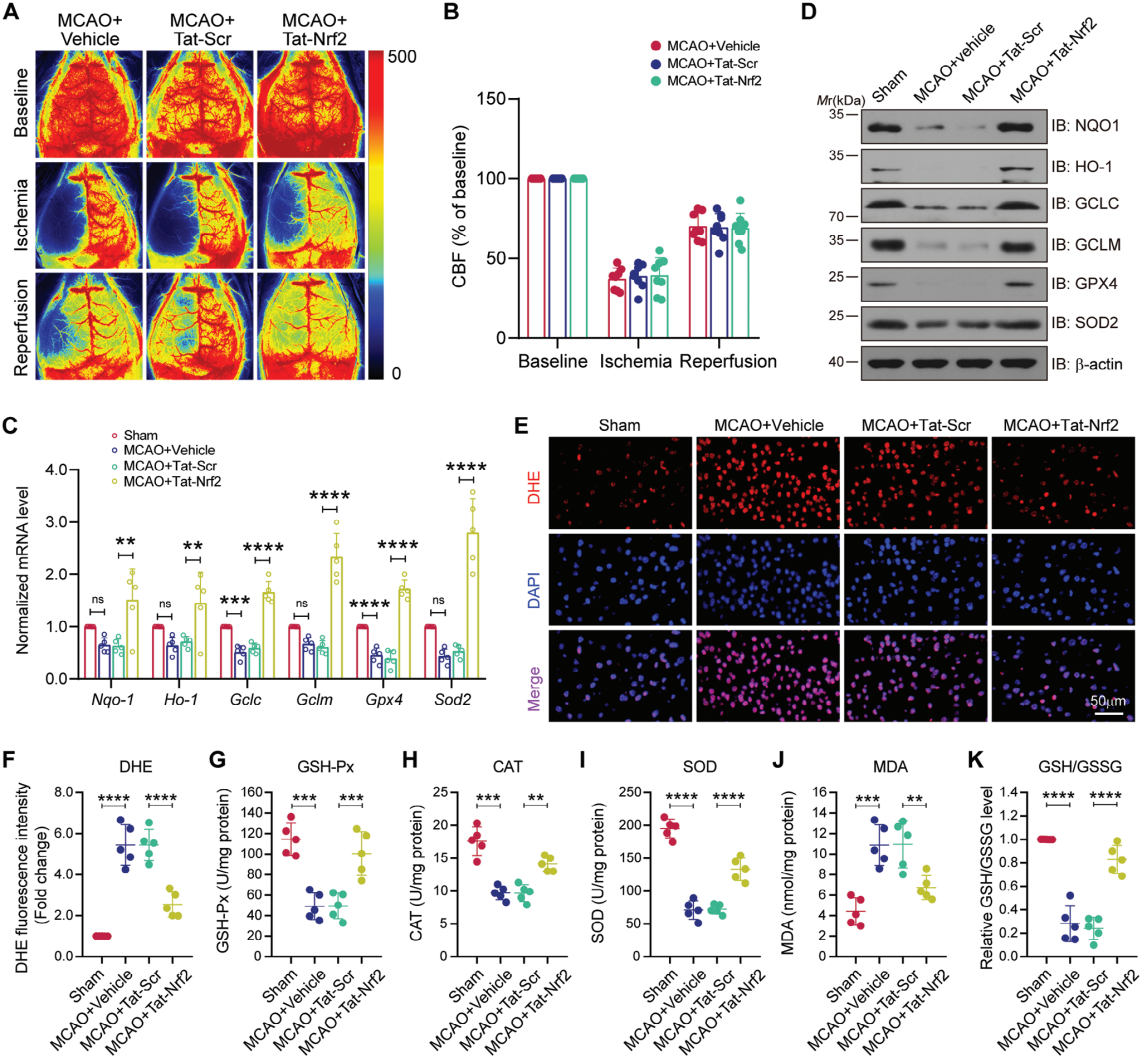
Tat‐Nrf2 peptide inhibits oxidative stress after ischemic stroke in mice. Mice were operated with sham or MCAO surgery and then injected (i.v.) with vehicle, Tat‐Nrf2 or Tat‐Scr peptide at 6 h following reperfusion at a dose of 10 mg kg^−1^ for 3 consecutive days. A) 2D laser speckle imaging techniques were applied to assess cerebral blood flow (CBF), showing successful ischemia and reperfusion in the left hemisphere. B) Quantitative analyses of rCBF changes in (A). C) qPCR was applied to examine the mRNA levels of *Nqo‐1*, *Ho‐1*, *Gclc*, *Gclm*, *Gpx4* and *Sod2*. D) Immunoblot analysis was applied to detect the protein level of total NQO1, HO‐1, GCLC, GCLM, GPX4 and SOD2. E) Dihydroethidium (DHE) staining to determine the production of ROS induced by ischemic stroke. Scale bar = 50 µm. F) Statistical analysis of the fluorescence intensities of DHE. G‐K) The results showing the effects of Tat‐Nrf2 peptide treatment on the activity of GSH‐Px, CAT, SOD, MDA and GSH/GSSG levels. Data are expressed as the mean ± SD and quantified by two‐way ANOVA followed by Tukey's post hoc test (B) or one‐way ANOVA followed by Dunnett's post hoc test (C, F‐K). n = 5 or 8 mice per group.; **p < 0.01, ***p < 0.001 and ****p < 0.0001.

To examine the neuroprotective effects of Tat‐Nrf2 peptide in mice subjected to focal ischemic stroke, we first monitored cerebral blood flow (CBF). During MCAO surgery and reperfusion, the mice administered Tat‐Nrf2 or Tat‐Scr peptide displayed equivalent regional CBF (**Figure** **7**A,B). Then, 72 h following ischemic insult and peptide injection, the expression of the Nrf2‐targeted antioxidant gene were determined. The data revealed that the mRNA expression of *Nqo‐1*, *Ho‐1*, *Gpx1*, *Gpx4*, *Gclc*, *Gclm*, *Sod1* and *Sod2* decreased after MCAO. However, this phenomenon was dramatically reversed by Tat‐Nrf2 treatment (Figure 7C). Immunoblot assays also confirmed these results (Figure 7D). In addition, we evaluated the fluorescence intensity of dihydroethidium (DHE). The findings indicated that Tat‐Nrf2 treatment notably decreased the fluorescence intensity of DHE induced by MCAO (Figure 7E,F). Furthermore, we compared the overall redox state. As shown in Figure 7G, Tat‐Nrf2 treatment significantly increased the GSH/GSSG ratio compared with Tat‐Scr. Finally, we assessed the activation of GSH‐Px, CAT, SOD and malondialdehyde (MDA). The results showed that Tat‐Nrf2 administration increased the activities of the antioxidant enzymes while decreasing the activities of MDA compared with Tat‐Nrf2 (Figure 7H,K). Collectively, these results suggest that Tat‐Nrf2 administration protects against oxidative stress injury after MCAO.

### 2.8 Tat‐Nrf2 Peptide Treatment Protects Against Ischemic Brain Injury In Vivo

**Figure 8:**
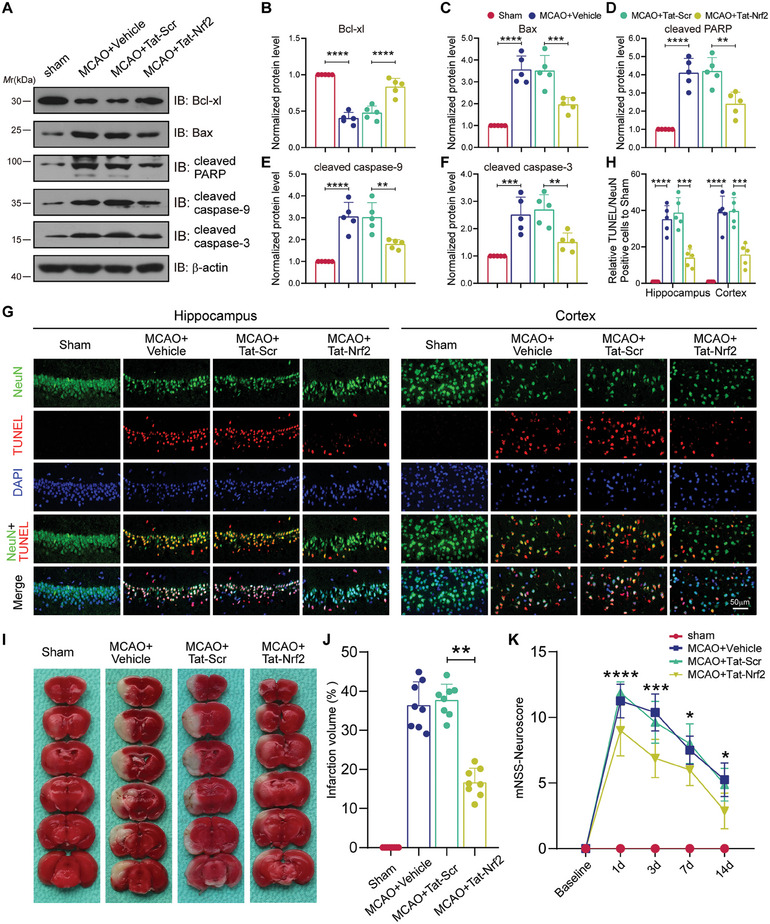
Tat‐Nrf2 peptide administration protects against ischemic brain injury in vivo. Mice were operated sham or MCAO surgery and then injected (i.v.) with vehicle, Tat‐Nrf2 or Tat‐Scr peptide at 6 h following reperfusion at a dose of 10 mg kg^−1^ for 3 consecutive days. A) Immunoblot was applied to determine the protein expression of Bcl‐xl, Bax, cleaved PARP, cleaved caspase‐9 and cleaved caspase‐3; n = 5 mice per group. B‐F) Statistical analysis of the indicated protein levels in (A). G) TUNEL staining was applied to determine the number of apoptotic cells in the peri‐infarct hippocampal and cortical tissues of mice. Scale bar = 50 µm. H) Statistical analysis of the number of NeuN‐ and TUNEL‐positive cells in (G). n = 5 mice per group. I) Tetraphenyl tetrazolium chloride (TTC) staining was applied to detect the infarct areas in mice 72 h after stroke. J) Quantitative analyses of infarct volumes in (I). K) The modified neurological severity score (mNSS) was conducted to detect the neurological deficiency at the indicated time after reperfusion; n = 8 mice per group. Data are expressed as the mean ± SD and quantified by one‐way ANOVA followed by Tukey's post hoc test (B‐F, H and J) or two‐way ANOVA followed by Tukey's post hoc test (K). *p < 0.05, **p < 0.01, ***p < 0.001 and ****p < 0.0001.

Next, we employed immunoblot assays to investigate the expression of apoptotic molecules in vivo. The results indicated that Tat‐Nrf2 administration reduced the protein level of proapoptotic molecules but increased the expression of antiapoptotic molecules (**Figure** **8**A–F). Then, 48 h following ischemic insult and peptide injection, TUNEL labeling was applied to evaluate neuronal death, and the results indicated that ischemic stroke led to an increase in apoptotic cells in both the hippocampus and cerebral cortex. However, the administration of Tat‐Nrf2 markedly decreased the number of apoptotic cells (Figure 8G,H). Furthermore, tetraphenyl tetrazolium chloride (TTC) staining was used to examine the extent of infarction following reperfusion for 48 h. The results demonstrated that the administration of Tat‐Nrf2 had a substantial effect on reducing the occurrence of cerebral infarction (Figure 8I,J). Finally, we employed a modified neurological severity score (mNSS) to explore neurological impairments at 1, 3, 7, and 14 days after reperfusion. The Tat‐Nrf2‐treated mice presented a better neurological score than the Tat‐Scr‐treated mice (Figure 8K). These data suggest that Tat‐Nrf2 peptide alleviates neuronal death, infarction, and neurological deficits after ischemic brain injury.

### 2.9 Tat‐Nrf2 Peptide Treatment Improves Motor and Cognitive Function After Ischemic Stroke In Vivo

**Figure 9:**
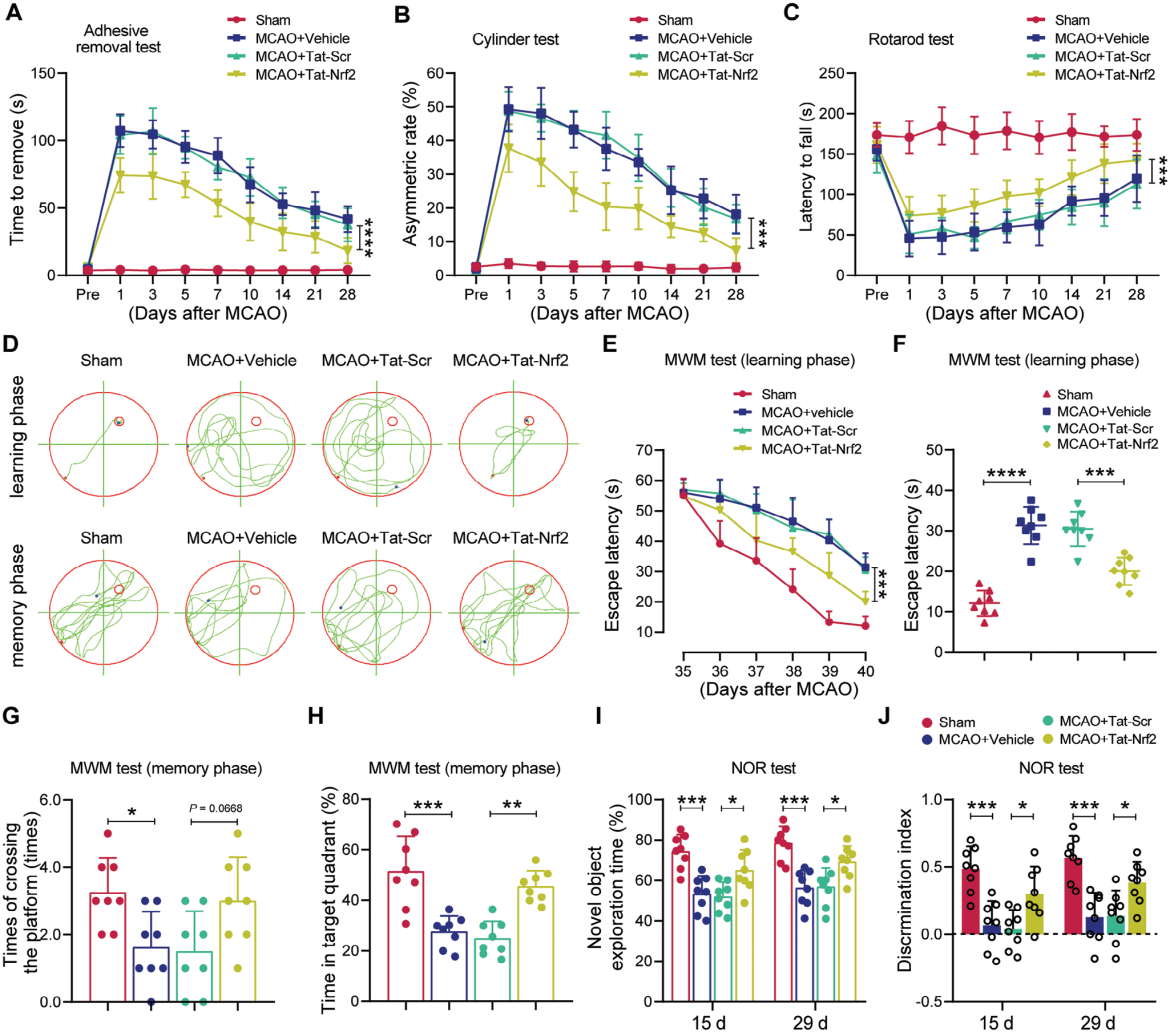
Tat‐Nrf2 improved motor and cognitive ability in mice after ischemic stroke. Mice were operated with sham or MCAO surgery and then injected (i.v.) with vehicle, Tat‐Nrf2 or Tat‐Scr peptide at 6 h following reperfusion at a dose of 10 mg kg^−1^ for 3 consecutive days. A‐C) A series of behavioral tests was conducted to determine sensorimotor dysfunction at the indicated time points after stroke. (A) Adhesive‐removal test. (B) Cylinder test. (C) Rotarod test. D‐H) The Morris water maze test was performed to determine the spatial learning and memory ability of mice. (D) Representative traces displaying the sample paths of the mice from the maze latency trials (learning) and the swimming traces from probe trials (memory). (E) The latency to the hidden platform as tested on Days 35–40 (defined as spatial learning). (F) The latency to the submerged platform as tested on Day 40. (G, H) Spatial memory was examined on Day 41 by evaluating the number of crossings of the original platform (G) and the time spent swimming in the target quadrant (H) after the platform was removed. I, J) Novel object recognition test, the exploration time of the mice in the familiarization phase (I), and the percentage of time exploring the novel object in the test phase (J). Data are expressed as the mean ± SD. Statistical differences in panels (A‐C and E) were analyzed by RM ANOVA followed by Tukey's post hoc test. Data in panel (G) were analyzed by the Kruskal–Wallis nonparametric test, data in panels (F, H) were analyzed by one‐way ANOVA followed by Tukey's post hoc test, and all others were analyzed by one‐way ANOVA followed by Tukey's post hoc test. n = 8 mice per group. *p < 0.05, **p < 0.01, ***p < 0.001 and ****p < 0.0001.

Next, we carried out adhesive removal test and cylinder test to examine sensorimotor function of mice after ischemic stroke. The results demonstrated that Tat‐Nrf2 administration facilitated neurological recovery, resulting in a decreased time for the mice to remove adhesive tape from their forepaws, as well as a reduced asymmetry rate in the cylinder test (**Figure** **9**A,B). Furthermore, rotarod tests were conducted to examine motor function. The findings demonstrated that Tat‐Nrf2 treatment considerably lengthened the duration spent on the rotating rods (Figure 9C). In addition, the Morris water maze (MWM) test was used to investigate memory deficits. Representative mouse route tracings from maze latency trials and the swimming traces from probing trials are presented in Figure 9D. We found that Tat‐Nrf2 administration significantly ameliorated memory impairments. The mice administered the Tat‐Nrf2 peptide spent less time to reach the submerged platform during the maze latency trials (Figure 9E,F). In addition, the mice treated with Tat‐Nrf2 exhibited a higher frequency of platform crossings and a longer duration spent in the target quadrant of the spatial probe trials in comparison to animals administered Tat‐Scr following ischemic stroke (Figure 9G,H). Finally, we explored mouse memory by the novel object recognition task. The results revealed that the mice administered the Tat‐Nrf2 peptide spent more time exploring novel objects compared to the mice treated with Tat‐Scr, demonstrating an improvement in declarative recognition memory after ischemic stroke (Figure 9I,J). In summary, these findings collectively suggest that Tat‐Nrf2 peptide administration enhances long‐term neurological function after ischemic stroke.

To further study Tat‐Nrf2 peptide as an ischemic stroke therapeutic candidate, the safety profile of Tat‐Nrf2 was investigated. Tat‐Nrf2 was injected into mice at a dose of 20 or 100 mg kg^−1^ daily for 7 consecutive days. The animals showed no behavioral evidence of toxicity after this treatment. In addition, no elevation of any liver transaminases or biomarkers of renal dysfunction was detected (Figure S12, Supporting Information). H&E staining revealed that no cerebral, cardiac, hepatocellular, renal, or pulmonic cellular damage was observed (Figure S13, Supporting Information), indicating that Tat‐Nrf2 induced little toxicity in mice.

## 3 Discussion

**Figure 10:**
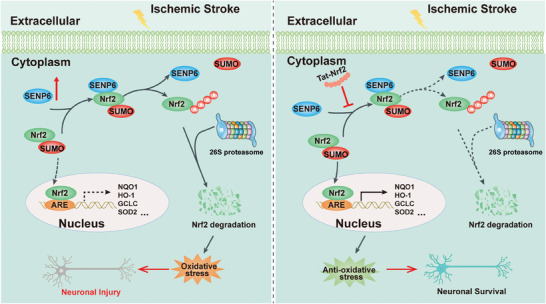
Scheme depicting the proposed mechanisms for the neuroprotective effects of Tat‐Nrf2 peptide treatment in ischemic stroke. Following ischemic stroke, Tat‐Nrf2 peptide treatment specifically blocked the binding of SENP6 with Nrf2 and inhibited SENP6‐mediated deSUMOylation of Nrf2, followed by blocking its ubiquitin proteasome‐dependent degradation, thus potentiating the Nrf2‐ARE signaling pathway, which promotes the expression of ARE‐regulated antioxidative genes, ultimately reducing oxidative stress and exerting anti‐ischemic neuroprotective effects.

In this study, we explored the effect of ischemic stroke‐trigged deSUMOylation of Nrf2 on neurons oxidative damage and the underlying mechanism. We provided evidence to support the notion that SENP6 acts as a sentrin/SUMO‐specific protease specifically involved in the deSUMOylation process of Nrf2. Interestingly, SENP6‐induced deSUMOylation of Nrf2 can decrease its protein stability and increase its ubiquitination modification, followed by degradation via the ubiquitin proteasome system. Subsequent investigations have demonstrated that deSUMOylation of Nrf2 facilitates the generation of ROS and lipid peroxidation. This process ultimately leads to increased oxidative stress and results in neuronal apoptosis, ischemic infarction, and impaired neurological function following ischemic stroke. Furthermore, this work also provided evidence that the use of a cell membrane‐permeable peptide to inhibit the deSUMOylation of Nrf2 effectively mitigates neuronal death, ischemic infarction, and alleviates neurological impairments following ischemic brain injury (working model shown in **Figure** **10**).

SUMOylation is one of the most important PTMs, which is similar to ubiquitination. Numerous evidence has indicated that SUMOylation plays crucial roles in regulating the various functions of the target protein.^[^ ^23^ ^]^ A previous study has demonstrated that Nrf2 can be conjugated by SUMO2 at lysins 110 and 533, and the SUMOylation of Nrf2 regulates its transcriptional activity.^[^ ^16^ ^]^ Guo et al. also showed that K110 in human Nrf2 is a bona fide SUMO‐acceptor for SUMO‐1 and has a role in serine biosynthesis in hepatocellular carcinoma.^[^ ^29^ ^]^ Consistent with these reports, we demonstrated that Nrf2 was predominantly modified by SUMO2/3 compared with SUMO1. Furthermore, our results also suggested that ischemic stroke induced deSUMOylation of Nrf2 mainly occurred at lysine residue 533. Numerous studies have demonstrated the pivotal roles of SUMOylation in controlling the stability of targeted proteins.^[^ ^15^ ^]^ In a previous study, it was demonstrated that RNF4 mediates polyubiquitination of SUMOylated Nrf2, resulting in its subsequent degradation in PML nuclear bodies.^[^ ^22^ ^]^ However, there is a report also claimed that SUMOylation of Nrf2 at K110 and K533 are in part needed for the stability of human Nrf2 in the nucleus.^[^ ^16^ ^]^ In this study, we demonstrated that the deSUMOylation of Nrf2 obviously enhanced its polyubiquitination, resulted in its degradation through the ubiquitin‒proteasome system, which suggested that the SUMOylation modification modulates the protein stability of Nrf2 in neurons after ischemic stroke.

Growing evidence has demonstrated that Nrf2 plays a crucial role in maintaining redox homeostasis and may hold promise as a therapeutic target for resolving ischemic stroke.^[^ ^30^ ^]^ Zhou et al. found that deSUMOylation of Nrf2 in laryngeal carcinoma is involved in the inhibition of the Nrf2/ARE pathway after cisplatin‐induced ROS stress.^[^ ^31^ ^]^ However, whether and how Nrf2 SUMOylation influences neuronal fate in the brain during ischemic stroke remains poorly understood. In this study, we found that the deSUMOylation of Nrf2 reduce its nucleus translocation and transcriptional activity, block antioxidant enzyme expression, induce oxidative stress damage and aggravate neuronal damage after ischemic stroke. Recent findings also suggest that PTMs, such as acetylation,^[^ ^32^ ^]^ ubiquitination^[^ ^33^ ^]^ and phosphorylation,^[^ ^34^ ^]^ strictly modulate the activation of Nrf2. The interaction of numerous PTMs, which alter protein function synergistically or antagonistically, has received growing attention.^[^ ^35^ ^]^ In previous research, we demonstrated that SENP6 induced ANXA1 deSUMOylation can increase its serine phosphorylation.^[^ ^20^ ^]^ Therefore, further investigation is needed to determine whether cross‐talk exists between Nrf2 phosphorylation, acetylation, ubiquitination and SUMOylation remains to be systematically clarified.

The SUMO protease SENP6 prefers SUMO2/3‐modified substrates and participates in the proteolytic removal of SUMO chains from substrates.^[^ ^36^ ^]^ SENP6 paly critical roles in the control of genome stability, cell division, neuronal apoptosis and neuroinflammation,^[^ ^37^ ^]^ but whether and how SENP6 has an effect on oxidative stress remains poorly understood. The present work reveals that SENP6 acts as a distinct isopeptidase to remove SUMO2 chains from Nrf2. These findings support the general notion that Nrf2 preferentially might be possible target for SUMO2 modification.^[^ ^16^ ^]^ In this study, we found that SENP6 binds to Nrf2 and OGD/R significantly enhances this binding. There are a number of possible explanations for this phenomenon. One is simply that SENP6 itself undergoes some kind of post‐translational modification and its enzymatic activity is enhanced, resulting in increased binding to Nrf2 after OGD/R. An alternative possibility is that the protein structure of SENP6 and/or Nrf2 is altered under OGD/R conditions, making it easier for the two to bind and thus increasing SENP6‐Nrf2 binding. Of course, these hypotheses need to be tested in further studies.

In a previous study, Zhou et al. demonstrated that nuclear Nrf2 activity in laryngeal carcinoma is regulated by SENP3 after cisplatin‐induced oxidative stress,^[^ ^31^ ^]^ but in this study, we found that SENP6, rather than SENP3, effectively bound to Nrf2 and deconjugate SUMO2 from the Nrf2 protein. Despite the fact that deSUMOylation of Nrf2 by SENP6 had little impact on the expression level of antioxidant enzyme genes under normal condition, these processes may be important in preserving a balance between the level of Nrf2 SUMOylation under normal condition, as SUMOylation is typically dynamic and reversible. Additionally, upregulation of SENP6 reduced the Nrf2 SUMOylation level, inhibited Nrf2/ARE signaling pathway dependent antioxidant response and eventually leading to neuronal apoptosis after ischemic stroke. Reversely, our further research revealed that downregulation of SENP6 by a specific shRNA significantly alleviates oxidative stress and neuronal damage after OGD/R. The present research confirmed that SENP6 promoted neuronal oxidative stress induced by cerebral I/R injury.

Cell membrane‐permeable peptides have been used in preclinical and clinical trials to study fundamental biological concerns.^[^ ^38^ ^]^ Tat is a cell membrane‐permeable peptide that has a high efficiency for rapidly and efficiently translocating a variety of proteins, peptides, DNA and RNA cross the plasma membrane and blood‒brain barrier following noninvasive systemic administration.^[^ ^39^ ^]^ Numerous Tat fusion peptides have shown therapeutic effects in a variety of disease animal models and, in some cases, have been translated to clinical studies.^[^ ^40^ ^]^ It has recently been proven through the success of a phase 3 clinical study that a Tat‐fused short peptide, Nerinetide, also known as NA‐1, is not only safe but also therapeutically effective in preventing ischemia damage to human neurons.^[^ ^41^ ^]^ In the present study, we designed a series of peptides of varying lengths that exhibit overlap with the deSUMOylation site of Nrf2 and identified the Tat‐Nrf2 peptide can efficiently block the interaction between SENP6 and Nrf2. Moreover, we presented solid evidences that blocking Nrf2 deSUMOylation with Tat‐Nrf2 peptide showed robust neuroprotection against ischemic stroke. It is known that there are many other substrates of SENP6, such as BRCA1,^[^ ^18a^ ^]^ ANXA1,^[^ ^21^ ^]^ M18BP1,^[^ ^42^ ^]^ SLX4,^[^ ^43^ ^]^ which mediate various of biological processes including neuronal apopotosis, angiogenesis, and DNA repair. The application of Tat‐Nrf2 peptide could specifically block the binding of SENP6 to Nrf2, followed by inhibiting deSUMOylation of Nrf2 without affecting other substrates. We hope that this Tat‐Nrf2 peptide can also be successfully translated to the clinic as a new and effective therapeutic candidate for the cure of ischemic stroke in human patients. In this study, we reported that application of Tat‐Nrf2 result in effective delivery of the peptide into brain tissue and that administration within 6 h of stroke produced a therapeutic effect, whereas the therapeutic time window for tPA was < 4.5 h after stroke onset.^[^ ^44^ ^]^ Nonetheless, more detailed works are required to determine the exact therapeutic time window. As the Tat‐Nrf2 peptide provides effective ischemic neuroprotection when injected after reperfusion, it is also necessary to assess its effect when applicated prior to reperfusion.

There are still several limitations in this investigation. First, the pharmacokinetics parameters of Tat‐Nrf2 have not been determined. Therefore, more pharmacokinetic and pharmacodynamic studies in relevant animal models are needed to clarify the optimal dosage, bioavailability and half‐life of Tat‐Nrf2. Second, we only examined an ischemia‒reperfusion model in mice since peptides required blood flow to be transferred to the site of damage. There is currently no evidence on how damage might be affected if reperfusion cannot be restored. The protective effect of the Tat‐Nrf2 peptide on permanent occlusion models needs to be further studied. Third, unlike stroke patients, the mice employed in our stroke model are relatively young and lack many comorbidities. This may make the estimation of direct conversion efficacy challenging and may make it difficult to translate estimates of therapeutic efficacy directly. Furthermore, no clinical samples were available, so the results may be biased. Although this reduces variability and improves the ability of laboratory research to identify treatment effects, it does not provide information on patient response to the numerous types of strokes observed clinically or the expected variability of response in clinical studies. Finally, only male rats were evaluated in the in vivo studies. It remains unclear whether the function of the Tat‐Nrf2 peptide differs by sex, and further investigation is needed. Such limits should be considered while developing clinical studies.

In conclusion, our study has provided convincing evidence that SENP6 exerts as a SUMO‐specific isopeptidase to induce Nrf2 deSUMOylation, thereby upregulating its ubiquitination modification and promoting its degradation via the ubiquitin proteasome system, resulting in inhibiting the Nrf2‐dependent antioxidant response, ultimately leading to oxidative stress and neuronal death after ischemic stroke. Furthermore, the in vivo evidence revealed that the disruption of the binding between SENP6 and Nrf2 using the Tat‐Nrf2 peptide leads to a significant decrease in brain injury and alleviated neurological outcomes in mice after ischemic stroke. This positive effect is primarily attributed to the antioxidant properties of the Tat‐Nrf2 peptide in neurons, achieved by enhancing Nrf2 activation through the inhibition of SENP6‐mediated deSUMOylation. Overall, the aforementioned data revealed a previously undefined role of SENP6 and indicated that inhibiting the activity of SENP6 or its binding with Nrf2 would be a novel and promising therapeutic strategy for ischemic stroke and possibly other neurological disorders characterized by oxidative stress manifestations.

## 4 Experimental Section

### Animals

Adult male C57BL/6 mice (8–12 weeks) were obtained from the Charles River (Beijing Office, China). Following their arrival, the animals had a period of acclimatization lasting one week, during which they were housed in a controlled environment free from any pathogens. Mice were housed in polycarbonate cages and provided with standard sawdust bedding. They were maintained in controlled conditions where the temperature was maintained at 22 ± 2 °C, humidity at 55 ± 10%, and subjected to alternating light and dark cycles with a 12‐h duration (lights were on from 8:00 to 20:00 h). The subjects were provided with regular laboratory food and were granted unrestricted access to water as needed. The food underwent a radioactive treatment, the water was subjected to filtration, and the cage equipment was sterilized. All scientific investigations pertaining to animals were performed in compliance with the Animal Research: Reporting of In Vivo Experiments (ARRIVE) guidelines. All experimental protocols received approval from the Experimental Animal Care and Use Committee of Tongji Hospital, Tongji Medical College, Huazhong University of Science and Technology (TJH‐2020050025). The allocation of animals to each group was chosen based on reported numbers from published research or our previous experiment, and the precise number of animals was included in the figure legends. The animals were randomly divided into different groups by a random number generator. The experiments were conducted by researchers who were unaware of the group assignments.

### Antibodies and Reagents

The antibodies used in the current study are listed in Table S1 (Supporting Information). The Protein A+G agarose beads were acquired from Beyotime Biotechnology (Shanghai, China). The Ni^2+^‐NTA agarose was procured from Qiagen (Dusseldorf, Germany). The CHX compound was acquired from Calbiochem (508739; Darmstadt, Germany). Dimethyl sulfoxide (DMSO; D2650), MG‐132 (C‐2211), ammonium chloride (NH_4_Cl; A9434), 3‐MA (M9281), and CQ phosphate (PHR1258) were obtained from Sigma–Aldrich (Shanghai, China). The entire protease inhibitor cocktail as well as the In Situ Cell Death Detection Kit were purchased from Roche (Basel, Switzerland). All other generic reagents were obtained from commercial sources and used exactly as received.

### Transient Focal Cerebral Ischemia

The transient MCAO model was performed in male mice as previously described.^[^ ^45^ ^]^ Briefly, the animals were first given 2.5% isoflurane (RWD Life Science, Shenzhen, China) anesthesia. Subsequently, a midline cervical incision was made to surgically expose the left common carotid artery (CCA), internal carotid artery (ICA), and external carotid artery (ECA). The CCA was ligated using a live ligature, while the ECA was ligated using two monofilaments. One monofilament was ligated to the distal end of the ECA, while the second monofilament was ligated to both the external and internal carotid artery branches. Ultimately, an interventional procedure was performed wherein an opening was established between the ECA ligatures. Subsequently, a 4‐0 nylon suture, characterized by a diameter of 0.25 ± 0.03 mm and equipped with a silicon tip, was carefully inserted through the ECA stump and directed toward the ICA. This maneuver effectively resulted in occlusion of the MCA. The Laser Speckle Imaging System (RFLSI III, RWD Life Science) was utilized to obtain the CBF. After a duration of 1 h of MCAO, the suture was removed to initiate reperfusion. The experimental group of mice underwent the identical surgery, with the exception that embolus inserts were not included.

### Cell Culture, Transfection and OGD/R

HEK293T cells were acquired from the American Type Culture Collection. Cells were cultured at 37 °C in a humidified 5% CO_2_‐containing environment. The plasmids were transduced into cells by Lipofectamine 3000 (Invitrogen, Waltham, USA) when the cells were 80 to 90% confluent. Primary neurons were dissected from the mouse embryos (E16) following a previous method.^[^ ^46^ ^]^ Briefly, under a dissecting microscope, the cerebral cortex was gently isolated and then digested with 0.125% trypsin for 5 min, followed by filtration through a 70 µm pore size filter. Following washing with PBS, the collection of the single‐cell mixture was performed using Dulbecco's Modified Eagle Medium (DMEM, Thermo Fisher Scientific, Waltham, USA) supplemented with 10% FBS (Gibco, Gaithersburg, USA). The medium was replenished every 72 h with neurobasal medium supplemented with 2% B27 (Gibco) and 1% GlutaMAX (Thermo Fisher Scientific). The OGD/R was performed as previously described.^[^ ^47^ ^]^ Prior to OGD induction, the supernatant of cultured cells was replaced with glucose‐free DMEM media (Gibco). Subsequently, the cells were cultivated at a temperature of 37 °C within an anaerobic incubator, wherein an environment consisting of 1% O_2_, 94% N_2_, and 5% CO_2_ was maintained. Following a duration of 60 min, the cells were subjected to cultivation using a normal medium and subsequently returned to the normal incubator to undergo reoxygenation.

### Ni^2+^‐NTA Pull‐Down Assay

Ni^2+^‐NTA pull‐down was applied to purify the SUMOylated Nrf2 after denaturation as described previously.^[^ ^48^ ^]^ HEK293T cells were treated with 800 µl Ni^2+^‐NTA denatured buffer, which consisted of a solution containing 10 mM Tris, 20 mM NEM, 6 M Gu‐HCl, and 100 mM NaH_2_PO_4_ at a pH of 8.0 after being rinsed twice with cold PBS. After the DNA was sliced by ultrasound treatment (2 × 20 s), the sample was cleaned using centrifugation at 15 000 g, and 4 °C for 10 min. The supernatant was mixed with 50 µL of pre‐washed Ni^2+^‐NTA agarose (Qiagen) and incubated for 3 h on a shaker at 4 °C. The beads were then washed in 1 mL of Ni^2+^‐NTA washing solution (0.1% Triton X‐100; 100 mM NaH_2_PO_4_; 10 mM Tris/HCl, pH 6.3; 8 M Urea) before being loaded into 50 µL of 2 × SDS‐PAGE loading buffer with 200 mM imidazole at 95 °C for 5 min. Finally, the proteins were separated by SDS–PAGE and analyzed by immunoblot.

### Immunoblot

The cells were lysed using ice‐cold RIPA buffer obtained from Beyotime Biotechnology. The lysates underwent centrifugation and were then combined with gel‐loading buffer. The mixture was then subjected to boiling at a temperature of 95 °C for 5 min. The samples underwent resolution by 10% or 12% SDS‐PAGE. Subsequently, the resolved samples were transferred onto a PVDF membrane (Merck‐Millipore, Billerica, USA). The membranes were then blocked using a 5% fat‐free milk solution and then incubated overnight at a temperature of 4 °C with the primary antibodies. After washing three times with TBST, the bound primary antibody was detected using either anti‐mouse IgG or anti‐rabbit IgG (Beyotime Biotechnology). Finally, the antibody‐bound proteins were detected by a chemiluminescence substrate kit (ECL; Advansta, San Jose, USA). The data were analyzed with ImageJ software. The relative concentrations of these proteins were adjusted to *β*‐actin. The quantification data of the immunoblots were shown in the corresponding figures or Figure S14 (Supporting Information).

### Co‐Immunoprecipitation (Co‐IP)

Co‐IP assay was performed as previously reported.^[^ ^49^ ^]^ In brief, cellular lysis was conducted using immunoprecipitation (IP) buffer obtained from Beyotime Biotechnology. The IP solution was supplemented with complete protease inhibitor cocktail tablets (5 mg mL^−1^; Roche, Basel, Switzerland). The lysis process was carried out at a temperature of 4 °C for 15 min. Then the extracted sample was subjected to centrifugation with 14 000 ×g for 15 min. Subsequently, the supernatant was collected and used for further analysis. The samples were incubated overnight on a revolving rocker with the specified primary antibodies. Then, protein A/G agarose (Beyotime Biotechnology) was added and incubated for 4 h. Subsequently, the samples underwent a gentle rinsing process using PBS three times. They were then supplemented with gel‐loading buffer and then boiled at a temperature of 95 °C for 8 min. Finally, the samples were processed for further immunoblot analysis.

### TUNEL Staining

Cell apoptosis in frozen mouse brain slices was determined by TUNEL staining following the instructions provided by the manufacturer (Roche). The tissue sections were stained and then sealed with mounting solution containing DAPI (Sigma–Aldrich). Afterward, fluorescence microscopy (BX53, Olympus, Tokyo, Japan) was used to capture images. To mitigate bias, a minimum of four microscopic fields were captured inside the ischemic penumbra of each segment. The TUNEL cells in each field were counted in five sections from each mouse by an investigator who was unaware of the research design. The average number of TUNEL cells in the fields of view was then calculated for each animal.

### DNA‐Binding Activity Assays

Nuclear extracts were generated from primary neurons using a nuclear extract kit (Active Motif, Carlsbad, USA). The DNA‐binding activity of Nrf2 was evaluated using the TransAM Nrf2 kit (Active Motif), which includes an immobilized ARE consensus binding site (5′‐GTC ACA GTG ACT CAG CAG AAT CTG‐3′). The experimental procedure was conducted according to the manufacturer's instructions. In summary, a total of 40 µL of complete binding buffer was added, followed by the addition of 20 µg of nuclear extract samples. The plate was subjected to coverage and agitation for a duration of 1 h at room temperature. The oligonucleotides exhibited specific binding affinity toward the protein in its activated form, which was then detected using the provided primary antibody and secondary antibody coupled with horseradish peroxidase. The assessment of the transcription factor activity involved the measurement of absorbance at 450 nm using a spectrophotometer. This measurement was taken following the addition of a developing solution and the subsequent cessation of the colorimetric reaction.

### Measurement of Lipid Peroxidation and Antioxidant Enzymatic Activities

The amount of lipid peroxidation was assessed using the MDA testing. The determination of MDA content was conducted using commercially available kits (Beyotime Biotechnology) in accordance with the procedures provided by the assay kits. The absorbance of the supernatant at a wavelength of 532 nm was examined using spectrophotometry. The MDA concentration was computed using the standard curve and thereafter expressed as micromoles per milligram of protein. The cells were collected using PBS, subjected to sonication while kept on ice, and subsequently centrifuged at 4 °C. The measurement of antioxidant enzyme activity was conducted using supernatant lysates obtained from primary cultured neurons. The endogenous scavengers of ROS, SOD and GSH‐Px were assessed in brain tissue by the utilization of commercially available assay kits (Cayman Chemical, Ann Arbor, USA), following the directions provided by the manufacturer. The levels of SOD were quantified as units/mg protein, and the activity of SOD was assessed by its capacity to inhibit the production of superoxide radicals produced by the xanthine oxidase system. The activity of GSH‐Px was assessed following the guidelines provided by the manufacturer. In summary, the enzyme glutathione reductase (GR) was employed in a connected procedure to assess the activity of GSH‐Px. The oxidized form of GSSG, which was generated by the enzyme GSH‐Px, was then reduced by the actions of GR and NADPH. NADPH was converted to NADP^+^ by GR, which reduced absorbance at 340 nm. The unit of GSH‐Px was defined as the amount of enzyme necessary to facilitate the oxidation of 1.0 mmol of NADPH to NADP^+^ per minute at 25 °C. The activities of GSH‐Px in the brain samples were expressed as U/mg protein.

### Measurement of Half‐Life of Nrf2

Recombinant wild‐type or mutant HA‐Nrf2 were generated to test the stability of Nrf2. HA‐Nrf2‐K533R mutant or wild‐type HA‐Nrf2 expression plasmids were transfected into HEK293T cells using lipofectamine 3000. 48 h later, the cells were exposed to cycloheximide (100 µg mL^−1^) for a variety of time periods, up to a maximum of 9 h. Then the cells were washed with PBS and lysed using a non‐denaturing lysis solution at the end of each time point. The whole cell lysate was electrophoresed on SDS‐PAGE, and then the levels of Nrf2 were determined using immunoblot by an anti‐HA antibody.

### DHE Staining

DHE staining was conducted to examine ROS levels induced by ischemic stroke. In brief, frozen brain slices measuring 10 µm in thickness were placed in a chamber containing humidity for 30 min. During this time, the fluorescent dye DHE (D1168, Thermo Fisher Scientific, Waltham, USA) was added to the chamber at a concentration of 2 µM. The temperature of the chamber was maintained at 37 °C. Using ImageJ software (NIH, Bethesda, USA), DHE‐positive cells were counted under a fluorescence microscope (BX53, Olympus).

### Real‐Time Quantitative PCR Analysis

The isolation of total RNA from primary cultured neurons was performed using TRIzol reagent (Invitrogen, Carlsbad, USA) according to the prescribed procedure.^[^ ^47^ ^]^ Afterward, spectrophotometric analysis was employed to evaluate the purity of the RNA. Following the manufacturer's instructions, complementary DNA was synthesized using the ReverTra Ace‐α‐TM First Strand cDNA Synthesis Kit (Toyobo, Osaka, Japan). The StepOnePlus Real‐Time PCR System (Applied Biosystems, Carlsbad, CA, USA) was used to perform qPCR. The 2^−ΔΔCt^ method was applied to access gene expression, and considering their amplification efficiencies, the relative expression ratios of target gene were calculated. The primers for this study are listed in Table S2 (Supporting Information).

### Immunofluorescence

The 4% paraformaldehyde‐fixed primary neurons were carefully washed with PBS, and then subjected to 10% Triton X‐100 for 10 min. Subsequently, the coverslips were blocked with 10% donkey serum. After 1 h, the coverslips were subjected to overnight incubation at 4 °C with the primary antibodies. Following three washes, the fixed coverslips were incubated with Alexa Fluor 488‐conjugated AffiniPure goat anti‐mouse IgG (H+L) and Alexa Fluor 594‐conjugated AffiniPure goat anti‐rabbit IgG (H+L) (Jackson Immuno Research) for 1 h at room temperature. The fluorescence was recorded using a fluorescence microscope (BX53, Olympus). The Image J software was utilized to measure a range of 50–100 cells in each well. The analysis was performed in a blinded manner, with investigators unaware of the experimental conditions.

### TTC Staining

Following anesthesia, the mouse brain was retrieved via decapitation for TTC staining as described previously.^[^ ^50^ ^]^ The lower brain stem and olfactory bulb were meticulously dissected apart. The brain tissues were rapidly frozen at −20 °C for 20 min before being cut into six slices with a 2 mm thickness. The tissues were then immersed in TTC solution for 30 min, followed by fixation in 4% paraformaldehyde buffer. The coloration of ischemic tissues was white, in contrast to the pink or red coloration exhibited by normal tissues. The images were acquired with a camera of exceptional quality. The estimation of the infarction area in the brain tissues was conducted utilizing ImageJ software.

### Plasmids and Viruses

The coding sequences for the mRNAs of SUMO1, SUMO2, and SUMO3 were cloned into the pcDNA3.1 plasmid (Thermo Fisher Scientific). The Flag‐tagged Ubc9 and ubiquitin constructs were generated by the process of cloning the whole cDNA sequences into the p3×Flag CMV14 vector (Sigma‐Aldrich). The HA‐tagged Nrf2 was generated by cloning the full‐length cDNA into pcDNA4/TO (Invitrogen). The Myc‐tagged SENP1, SENP2, SENP3, SENP5, SENP6, SENP7 were constructed via cloning the indicated cDNA into pcDNA 3.1 vector. For site‐directed mutagenesis, all mutants Nrf2‐K110R, Nrf2‐K533R, Nrf2‐K110/533R (Lysine‐to‐Arginine) and SENP6‐C1030S (Cysteine‐to‐Serine) were generated through the utilization of homologous recombination. This process was facilitated by using the Trelief SoSoo Cloning Kit (TSINGKE, Beijing, China) and synthetic oligonucleotides that contained the desired mutations at the corresponding positions, resulting in the generation of lysine‐arginine mutations. The human SENP6 shRNA plasmids used for transfection of HEK293T cells were obtained from GenePharma (Suzhou, China). The designated nucleotide sequence for the human SENP6 shRNA was 5′‐GAC AGA ACT AAC AGA AGA GAA‐3′. The confirmation of all constructions was achieved using DNA sequencing analysis, which was conducted by Sangon Biotechnology (Shanghai, China).

The generation of adenoviruses containing SENP6 coding sequence and shRNA duplexes that target mouse SENP6 was carried out by Vigene Biosciences (Jinan, China), as described in our previous publication.^[^ ^21^ ^]^ The design and verification process of the shRNA sequence were conducted in the following manner: The sequence mSENP6, 5′‐GGG CAA ATC TAC TCA GTG TAG‐3′, was provided. Serially diluted quantities of recombinant adenovirus were used to transfect primary neurons. It concluded that the ideal multiplicity of infection ranged from 50:1 to 100:1. Following a 48‐h viral infection, the neuronal cells were exposed to OGD/R and/or further treatments.

### Neurobehavioral Experiments

The neurobehavioral tasks were conducted by investigators who were unaware of the experimental group assignment. The animals received training prior to undergoing surgery. The assessment of sensorimotor function was conducted by neurobehavioral tests administered between post‐stroke days 3 and 28. Cognitive performance, on the other hand, was evaluated specifically on days 35 to 41 after the surgical procedure. The protocols for each behavioral test are given in further detail below.

### Neurological Score

Neurological deficient were assessed using the mNSS in the MCAO model animals as previously reported.^[^ ^51^ ^]^ At 1, 3, 7, and 14 days after the MCAO surgery, testing was conducted. The mNSS comprises a range of motor tests, including evaluations of muscle status and aberrant movements, as well as sensory tests that encompass visual, tactile, and proprioceptive assessments. Additionally, beam balance tests and reflex tests were administered. The mNSS was assigned a composite score ranging from 0 to 18. The magnitude of the damage escalates according to the rising values of the mNSS. The experiments were conducted by an observer who was blind to the experimental circumstances.

### Cylinder Test

The cylinder test was performed to examine forelimb use asymmetry as previously described.^[^ ^21^ ^]^ The number of times each forelimb or both forelimbs were utilized to support the body on the wall of the cylinder throughout the course of a 5‐min observation period was recorded for each mouse. The mice were individually placed within a transparent cylinder measuring 9 cm in diameter and 15 cm in height. Behind the cylinder, two mirrors were positioned to provide 360° visibility. The formula used to determine preference for the nonimpaired forepaw (left) was (left‐right)/(left + right + both) ×100%. Depending on the extent of the lesion, injured mice show a stronger preference for the left forepaw than undamaged mice do for either forepaw.

### Adhesive‐Removal Test

To evaluate forepaw sensitivity and motor deficits, the adhesive removal test was employed as previously described.^[^ ^21^ ^]^ The distal‐radial area of each forelimb's wrist was used to place two tiny sticky paper dots (3 × 3 mm^2^) as bilateral tactile stimuli. The animals were placed calmly in their cages. The time taken to extract each stimulus from the forelimbs was recorded during three consecutive daily sessions. There was a minimum 5‐min gap between each experiment. Mice received a three‐day training period before either the MCAO or sham procedures.

### Rotarod Test

The rotarod test was conducted to examine the animals' motor coordination.^[^ ^52^ ^]^ Briefly, the mouse was quickly positioned on the revolving rod while the rotor was speeding up (10 speeds between 4 and 40 rpm for 5 min). Mice that walked on the accelerated spinning pole for 300 s were recorded as walking survivors. The retention time was measured from getting on the pole to falling off. To establish stable baseline values, all animals were trained three times per day two days prior surgery. The mean retention time was observed.

### MWM Test

The MWM test was used to examine the long‐term cognitive function of mice.^[^ ^47^ ^]^ The device was a circular pool that was filled to a height of 25 cm with water added with titanium dioxide (diameter: 160 cm; height: 50 cm). A concealed platform, with a diameter of 10 cm and a depth of 1.5–2.0 cm, was positioned in one of the pool's four quadrants. Around the pool, prominent cues (such as wall plates, a door, and the observer himself) were put in fixed locations. Mice were given 2‐day acclimatization period before the concealed platform training began. The mice were put into the water during training at one of four random start points in each quadrant, facing the side wall. Each mouse was given 60 s to locate the platform on its own or be directed there before spending 15 s there to aid in learning and directional memory. During the first six days, four experiments were conducted, each including the release of mice from four different random release sites. To represent learning and memory, a 60‐s probe test was conducted following the last acquisition training, during which the platform was taken out of the tank. Animals were measured for both the amount of time spent in the target quadrant and time spent moving around the target platform. Training sessions and probing tests were monitored and analyzed using the SMART real‐time video tracking system.

### Novel Object Recognition Test

The animals were granted 5 min to habituate the arena (50 cm × 50 cm container) without objects one day prior to the test. Two same objects A and B were placed on opposite sides of the test area, and then, the mice reentered into the arenas and explored for 5 min. After 1 h, a new object C, which had the same material and size but different in shape, was replaced with one of the two objects, and the mice were explore the objects for 5 min. Between each habituation period, 70% ethanol was used to clean, and dry paper was used to wipe the arena and objects. In order to allow ethanol evaporation, at least 5 min were left. During the test, a video camera was positioned above the arena and recorded the behavior. Discrimination index was presented as follow: The discrimination index = (the time spent on exploring the novel object – the time spent on exploring the familiar object) / the time of spent on exploring the novel and the familiar object.

### Statistical Analysis

The quantitative analysis was performed using GraphPad Prism version 9.2.0 (GraphPad Software, San Diego, USA). Data were expressed as mean ± SD from at least three independent experiments. The researchers used the two‐tailed Student's t‐test to evaluate the differences between two groups. One‐ or two‐way ANOVA was used to analyze multiple groups mean differences, and a post hoc Dunnett's test or Tukey's post hoc test was then carried out. During the training period of the MWM test, which lasted six days, the escape latency was examined using the repeated‐measures (RM) ANOVA. The level of significance was set at *p* < 0.05. The abbreviation “ns” was used to indicate that a result was not statistically significant.

## Conflict of Interest

The authors declare no conflict of interest.

## Author Contributions

Q.X. and X.L. acquired the funding, conceived and designed the study. Q.X. conducted most animal experiments, stereotaxic surgery, molecular experiments and data analysis. M.X.Q. and G.F.Z. contributed to animal experiments and molecular experiments. H.J.Z., L.Z. and M.M. gave much valuable suggestions and helpful discussion during this study. L.Q.Z., X.Z. and Y.L.Z. helped with the data collection and analysis. Q.X. and X.L. wrote the paper and drew the figures. All authors have read and approved the final version of the manuscript.

## Supporting information



Supporting Information

## Data Availability

The data that support the findings of this study are available from the cor‐responding author upon reasonable request.

## References

[1] S. K. Feske , Am. J. Med. 2021, 134, 1457. 34454905 10.1016/j.amjmed.2021.07.027

[2] a) M. H. H. Balch , S. M. Nimjee , C. Rink , Y. Hannawi , J. Stroke 2020, 22, 159; 32635682 10.5853/jos.2019.02978 PMC7341014

[3] A. Chamorro , U. Dirnagl , X. Urra , A. M. Planas , Lancet Neurol. 2016, 15, 869. 27180033 10.1016/S1474-4422(16)00114-9

[4] Q. Z. Tuo , S. T. Zhang , P. Lei , Med. Res. Rev. 2022, 42, 259. 33957000 10.1002/med.21817

[5] H. Pawluk , R. Kołodziejska , G. Grześk , A. Woźniak , M. Kozakiewicz , A. Kosinska , M. Pawluk , E. Grzechowiak , J. Wojtasik , G. Kozera , Int. J. Mol. Sci. 2022, 23, 15625. 36555265 10.3390/ijms232415625 PMC9779811

[6] Y. Sun , X. Yang , L. Xu , M. Jia , L. Zhang , P. Li , P. Yang , Curr. Neuropharmacol. 2023, 21, 1405. 36453490 10.2174/1570159X21666221129100308 PMC10324331

[7] T. Suzuki , M. Yamamoto , Free Radic. Biol. Med. 2015, 88, 93. 26117331 10.1016/j.freeradbiomed.2015.06.006

[8] a) G. E. Mann , Free Radic. Biol. Med. 2014, 75, S1; 10.1016/j.freeradbiomed.2014.10.595 26461277

[9] A. Nakano‐Kobayashi , A. Canela , T. Yoshihara , M. Hagiwara , Proc. Natl. Acad. Sci. USA 2023, 120, e2303809120. 37549281 10.1073/pnas.2303809120 PMC10438385

[10] a) S. R. Kim , K. J. Seong , W. J. Kim , J. Y. Jung , Int. J. Mol. Sci. 2022, 23, 4004;35409364

[11] E. Petsouki , S. Ender , S. N. Sosa Cabrera , E. H. Heiss , Antioxidants 2023, 12, 1586. 37627580 10.3390/antiox12081586 PMC10451539

[12] Y. Kawai , L. Garduno , M. Theodore , J. Yang , I. J. Arinze , J. Biol. Chem. 2011, 286, 7629. 21196497 10.1074/jbc.M110.208173 PMC3045017

[13] K. Ha Kim , R. T. Sadikot , J. Yeon Lee , H. S. Jeong , Y. K. Oh , T. S. Blackwell , M. Joo , Free Radic. Biol. Med. 2017, 113, 48. 28939422 10.1016/j.freeradbiomed.2017.09.011 PMC5889093

[14] A. K. Jain , A. K. Jaiswal , J. Biol. Chem. 2006, 281, 12132. 16513647 10.1074/jbc.M511198200

[15] H. M. Chang , E. T. H. Yeh , Physiol. Rev. 2020, 100, 1599. 32666886 10.1152/physrev.00025.2019 PMC7717128

[16] T. S. Walters , D. J. McIntosh , S. M. Ingram , L. Tillery , E. D. Motley , I. J. Arinze , S. Misra , Cell. Physiol. Biochem. 2021, 55, 141. 33770425 10.33594/000000351 PMC8279473

[17] a) K. Kunz , T. Piller , S. Muller , J. Cell Sci. 2018, 131, jcs211904; 29559551 10.1242/jcs.211904

[18] a) L. A. Claessens , M. Verlaan‐de Vries , I. J. de Graaf , A. C. O. Vertegaal , Nat. Commun. 2023, 14, 5893; 37735495 10.1038/s41467-023-41623-w PMC10514054

[19] X. Liu , W. Chen , Q. Wang , L. Li , C. Wang , PLoS Pathog. 2013, 9, e1003480. 23825957 10.1371/journal.ppat.1003480 PMC3694847

[20] Q. Xia , M. Mao , Z. Zeng , Z. Luo , Y. Zhao , J. Shi , X. Li , Theranostics 2021, 11, 7450. 34158860 10.7150/thno.60277 PMC8210613

[21] M. Mao , Q. Xia , G. F. Zhan , Q. J. Chu , X. Li , H. K. Lian , Cell Biosci. 2022, 12, 113. 35869493 10.1186/s13578-022-00850-2 PMC9308285

[22] M. T. Malloy , D. J. McIntosh , T. S. Walters , A. Flores , J. S. Goodwin , I. J. Arinze , J. Biol. Chem. 2013, 288, 14569. 23543742 10.1074/jbc.M112.437392 PMC3656310

[23] Y. Yang , Y. He , X. Wang , Z. Liang , G. He , P. Zhang , H. Zhu , N. Xu , S. Liang , Open Biol. 2017, 7, 170167. 29021212 10.1098/rsob.170167 PMC5666083

[24] X. Li , Q. Xia , M. Mao , H. Zhou , L. Zheng , Y. Wang , Z. Zeng , L. Yan , Y. Zhao , J. Shi , Sci. Adv. 2021, 7, eabc5539. 33523920 10.1126/sciadv.abc5539 PMC7817101

[25] S. C. Chang , J. L. Ding , Biochim. Biophys. Acta. Rev. Cancer 2018, 1870, 165. 30318471 10.1016/j.bbcan.2018.08.002

[26] A. L. Eggler , E. Small , M. Hannink , A. D. Mesecar , Biochem. J. 2009, 422, 171. 19489739 10.1042/BJ20090471 PMC3865926

[27] Z. Sun , Y. E. Chin , D. D. Zhang , Mol. Cell. Biol. 2009, 29, 2658. 19273602 10.1128/MCB.01639-08 PMC2682049

[28] M. Thiruvengadam , B. Venkidasamy , U. Subramanian , R. Samynathan , M. Ali Shariati , M. Rebezov , S. Girish , S. Thangavel , A. R. Dhanapal , N. Fedoseeva , J. Lee , I. M. Chung , Antioxidants 2021, 10, 1859. 34942962 10.3390/antiox10121859 PMC8698417

[29] H. Guo , J. Xu , Q. Zheng , J. He , W. Zhou , K. Wang , X. Huang , Q. Fan , J. Ma , J. Cheng , W. Mei , R. Xing , R. Cai , Cancer Lett. 2019, 466, 39. 31546024 10.1016/j.canlet.2019.09.010

[30] M. Sandberg , J. Patil , B. D'Angelo , S. G. Weber , C. Mallard , Neuropharmacology 2014, 79, 298. 24262633 10.1016/j.neuropharm.2013.11.004 PMC3958930

[31] Z. Zhou , J. Xu , X. Bao , J. Shi , B. Liu , Y. Chen , J. Li , J. Cancer 2019, 10, 3427. 31293646 10.7150/jca.30318 PMC6603410

[32] J. J. Xu , J. Cui , Q. Lin , X. Y. Chen , J. Zhang , E. H. Gao , B. Wei , W. Zhao , Int. J. Cardiol. 2021, 342, 82. 34403762 10.1016/j.ijcard.2021.08.007

[33] X. Wang , T. Zhou , X. Yang , X. Cao , G. Jin , P. Zhang , J. Guo , K. Rong , B. Li , Y. Hu , K. Liu , P. Ma , A. Qin , J. Zhao , Adv. Sci. (Weinh) 2023, 10, e2204438. 36965071 10.1002/advs.202204438 PMC10190621

[34] T. Liu , Y. F. Lv , J. L. Zhao , Q. D. You , Z. Y. Jiang , Free Radic. Biol. Med. 2021, 168, 129. 33794311 10.1016/j.freeradbiomed.2021.03.034

[35] M. Hernandez‐Valladares , R. Wangen , F. S. Berven , A. Guldbrandsen , Curr. Med. Chem. 2019, 26, 5317. 31241430 10.2174/0929867326666190503164004

[36] S. J. W. van den Berg , L. E. T. Jansen , Front. Cell Dev. Biol. 2023, 11, 1193192. 37181753 10.3389/fcell.2023.1193192 PMC10172491

[37] a) S. Mitra , D. L. Bodor , A. F. David , I. Abdul‐Zani , J. F. Mata , B. Neumann , S. Reither , C. Tischer , L. E. T. Jansen , Nat. Commun. 2020, 11, 501; 31980633 10.1038/s41467-019-14276-x PMC6981222

[38] G. Guidotti , L. Brambilla , D. Rossi , Trends Pharmacol. Sci. 2017, 38, 406. 28209404 10.1016/j.tips.2017.01.003

[39] A. Bolhassani , B. S. Jafarzade , G. Mardani , Peptides 2017, 87, 50. 27887988 10.1016/j.peptides.2016.11.011

[40] a) Z. Fang , D. Wu , J. Deng , Q. Yang , X. Zhang , J. Chen , S. Wang , S. Hu , W. Hou , S. Ning , Y. Ding , Z. Fan , Z. Jiang , J. Kang , Y. Liu , J. Miao , X. Ji , H. Dong , L. Xiong , Sci. Transl. Med. 2021, 13, eabb6716; 34108252 10.1126/scitranslmed.abb6716

[41] M. D. Hill , M. Goyal , B. K. Menon , R. G. Nogueira , R. A. McTaggart , A. M. Demchuk , A. Y. Poppe , B. H. Buck , T. S. Field , D. Dowlatshahi , B. A. van Adel , R. H. Swartz , R. A. Shah , E. Sauvageau , C. Zerna , J. M. Ospel , M. Joshi , M. A. Almekhlafi , K. J. Ryckborst , M. W. Lowerison , K. Heard , D. Garman , D. Haussen , S. M. Cutting , S. B. Coutts , D. Roy , J. L. Rempel , A. C. Rohr , D. Iancu , D. J. Sahlas , et al., Lancet 2020, 395, 878.32087818

[42] H. Fu , N. Liu , Q. Dong , C. Ma , J. Yang , J. Xiong , Z. Zhang , X. Qi , C. Huang , B. Zhu , Cell Res. 2019, 29, 254. 30631152 10.1038/s41422-018-0139-y PMC6460436

[43] E. Alghoul , M. Paloni , A. Takedachi , S. Urbach , A. Barducci , P. H. Gaillard , J. Basbous , A. Constantinou , Mol. Cell 2023, 83, 1640. 37059091 10.1016/j.molcel.2023.03.021

[44] W. Hacke , M. Kaste , E. Bluhmki , M. Brozman , A. Davalos , D. Guidetti , V. Larrue , K. R. Lees , Z. Medeghri , T. Machnig , D. Schneider , R. von Kummer , N. Wahlgren , D. Toni , E. Investigators , N. Engl. J. Med. 2008, 359, 1317. 18815396 10.1056/NEJMoa0804656

[45] Q. Xia , S. Gao , T. Han , M. Mao , G. Zhan , Y. Wang , X. Li , J. Neuroinflammation 2022, 19, 301. 36517900 10.1186/s12974-022-02665-x PMC9753274

[46] X. Li , Y. Zhao , Q. Xia , L. Zheng , L. Liu , B. Zhao , J. Shi , Cell Death Dis. 2016, 7, e2356. 27584794 10.1038/cddis.2016.259 PMC5059862

[47] Y. Yu , Q. Xia , G. Zhan , S. Gao , T. Han , M. Mao , X. Li , Y. Wang , Cell Biosci. 2023, 13, 99. 37248543 10.1186/s13578-023-01056-w PMC10226213

[48] Q. Xia , M. Mao , G. Zhan , Z. Luo , Y. Zhao , X. Li , iScience 2023, 26, 106953. 37332598 10.1016/j.isci.2023.106953 PMC10272502

[49] K. Yao , Q. Mou , X. Lou , M. Ye , B. Zhao , Y. Hu , J. Luo , H. Zhang , X. Li , Y. Zhao , J. Neuroinflammation 2023, 20, 202. 37670386 10.1186/s12974-023-02886-8 PMC10481494

[50] Q. Xia , G. Zhan , M. Mao , Y. Zhao , X. Li , Exp. Mol. Med. 2022, 54, 180. 35217833 10.1038/s12276-022-00734-y PMC8894463

[51] X. Li , L. Zheng , Q. Xia , L. Liu , M. Mao , H. Zhou , Y. Zhao , J. Shi , Cell Death Differ. 2019, 26, 260. 29769639 10.1038/s41418-018-0116-5 PMC6329796

[52] Q. Xia , X. Li , H. Zhou , L. Zheng , J. Shi , Cell Death Dis. 2018, 9, 657. 29844306 10.1038/s41419-018-0686-7 PMC5974363

