# Supplementary material for: SENP6‐Mediated deSUMOylation of Nrf2 Exacerbates Neuronal Oxidative Stress Following Cerebral Ischemia and Reperfusion Injury
Source: Adv Sci (Weinh). 2024 Dec 24;12(7):2410410. doi: 10.1002/advs.202410410 (PMC11831438; doi:10.1002/advs.202410410)
Supplement: Supplementary file 1 — Supporting Information [file ADVS-12-2410410-s001.pdf]

ADVANCED SCIENCE
Open Access

Supporting Information

for *Adv. Sci.*, DOI 10.1002/advs.202410410

SENP6-Mediated deSUMOylation of Nrf2 Exacerbates Neuronal Oxidative Stress Following Cerebral Ischemia and Reperfusion Injury

*Qian Xia, Mengxin Que, Gaofeng Zhan, Longqing Zhang, Xue Zhang, Yilin Zhao, Huijuan Zhou, Lu Zheng, Meng Mao and Xing Li**

Supporting Information for

SENP6-mediated deSUMOylation of Nrf2 exacerbates neuronal oxidative stress following cerebral ischemia and reperfusion injury

Qian Xia[1], Mengxin Que[1], Gaofeng Zhan[1], Longqing Zhang[1], Xue Zhang[1], Yilin Zhao[1], Huijuan Zhou[2], Lu Zheng[3], Meng Mao[4], Xing Li[1]*

*Corresponding author. E-mail: lixing88@hust.edu.cn

This supporting information file includes:

Fig. S1. OGD/R increased the protein levels of SENP6, but decreased the protein level of Nrf2 in primary cultured neuron after OGD/R.

Fig. S2. Nrf2 interacts with SENP6 through the Neh1 domain.

Fig. S3. SENP6-mediated deSUMOylation of Nrf2 decreased its lysine acetylation.

Fig. S4. SENP6 enhances Keap1-Nrf2 binding.

Fig. S5. Overexpression of Nrf2 reversed SENP6-iduced oxidative stress damage and neurotoxicity after ischemic stroke.

Fig. S6. NAC treatment revised the promotive role of SENP6 on neuron cell death after OGD/R.

Fig. S7. Tat-Nrf2 peptide treatment effectively blocked the SENP6-Nrf2 interaction and upregulated the SUMOylation level of Nrf2 in vitro.

Fig. S8. Tat-Nrf2 peptide has little effect on the SENP6-ANXA1 interaction and the SUMOylation level of ANXA1.

Fig. S9. Tat-Nrf2 peptide was efficiently infused into the hippocampus and cerebral cortex regions.

Fig. S10. Tat-Nrf2 peptide treatment effectively blocked the SENP6-Nrf2 interaction and upregulated the SUMOylation level of Nrf2 in mice after ischemic stroke.

Fig. S11. Tat-Nrf2 peptide treatment decreased the ubiquitination level and promoted the nucleus translocation of Nrf2 in mice after ischemic stroke.

Fig. S12. Safety profile of Tat-Nrf2 peptide.

Fig. S13. HE staining images showing the morphology of heart, lung, liver, kidney, and brain in mice.

Fig. S14. Quantification of the western blotting data.

Table S1. Antibodies employed in this study.

Table S2. Primers used in this study.

Supplementary Figures

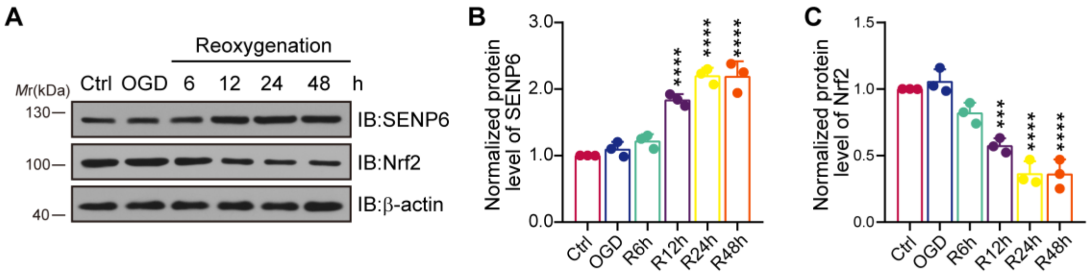

Fig. S1 OGD/R increased the protein levels of SENP6, but decreased the protein level of Nrf2 in primary cultured neuron after OGD/R. (**A**) Primary cultured neurons were treated with OGD and reoxygenation at the indicated time points. Whole cell lysates were subjected to immunoblots for the detection of SENP6 and Nrf2 protein expression. (**B**, **C**) The quantitative analysis of SENP6 and Nrf2 protein level. Data are reported as the mean ± SD from three independent experiments and analysed by one-way ANOVA followed by Dunnett's post hoc test. ***$P < 0.001$, ****$P < 0.0001$, compared with the control group.

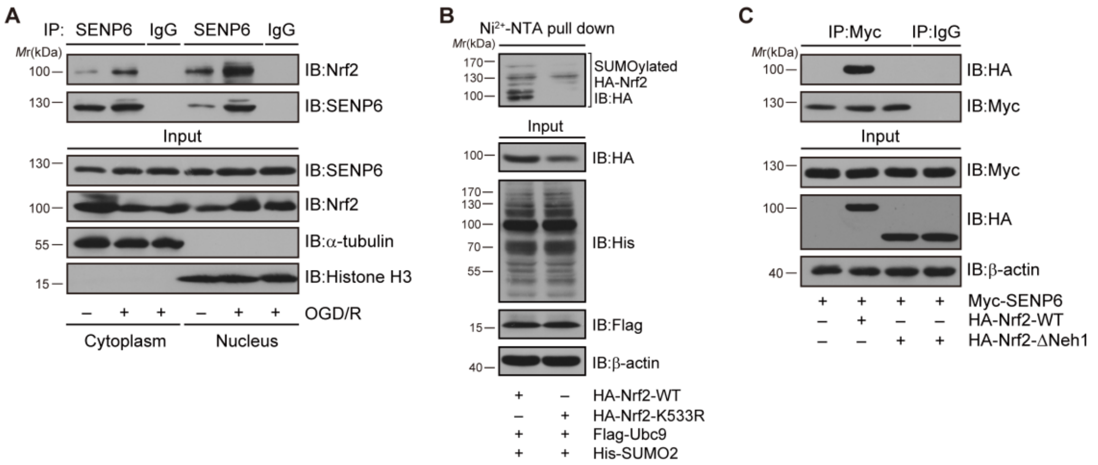

Fig. S2 Nrf2 interacts with SENP6 through the Neh1 domain. (**A**) Representative Co-IP results show the interactions of SENP6 with Nrf2 in the cytoplasm and nucleus in primary cultured neurons under normal or OGD/R conditions. (**B**) Representative Ni^{2+}-NTA pull-down assays results show the SUMOylation of wild-type Nrf2 and K533 mutant. (**C**) Representative Co-IP results show the interactions of full-length Nrf2 and the ΔNeh1 mutant construct with SENP6 in HEK293T cells. Data are representative of three independent experiments.

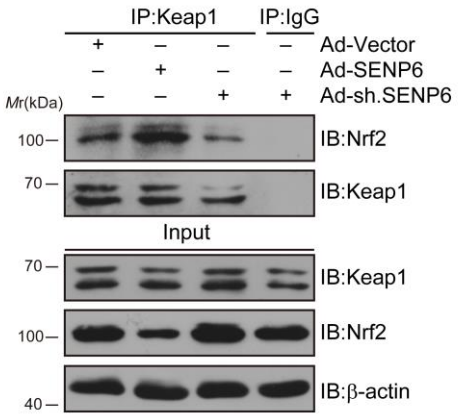

Fig. S3 SENP6 enhances Keap1-Nrf2 binding. Recombinant adenoviruses expressing the SENP6 coding sequence (Ad-SENP6), the SENP6 shRNA sequence (Ad-sh. SENP6) or the empty vector (Ad-vector) were infected into primary cultured neurons for 48 hours. Co-IP assays was performed to detected the endogenous binding between Keap1 and Nrf2.

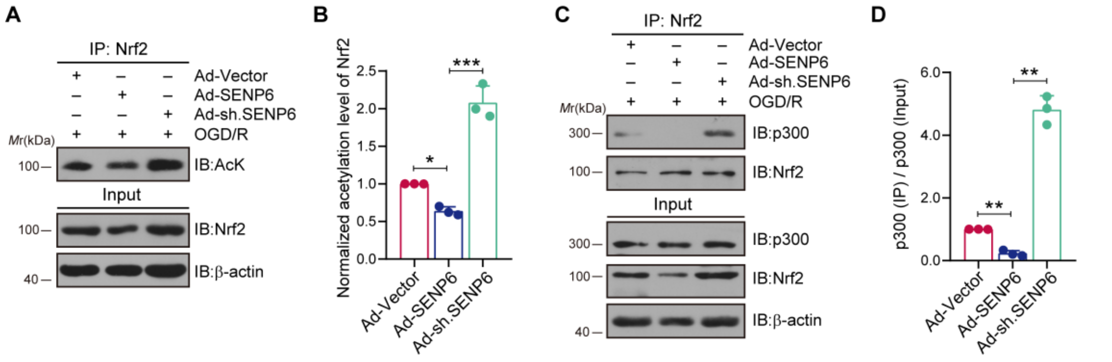

Fig. S4 SENP6-mediated deSUMOylation of Nrf2 decreased its lysine acetylation. (**A**) Primary cultured neurons were infected with adenovirus encoding SENP6-WT or SENP6 shRNA and were then subjected to OGD/R. Immunoprecipitation was conducted to examine the acetylation levels of Nrf2. (**B**) Quantification of the acetylation levels of Nrf2 shown in (A). (**C**) Primary neurons were infected with adenovirus encoding SENP6-WT or SENP6 shRNA and then subjected to OGD/R. Co-IP confirmed the interaction of endogenous p300 and Nrf2. (**D**) Quantification of the p300 binding with Nrf2. Data are reported as the mean ± SD from three independent experiments and analysed by one-way ANOVA followed by Dunnett's post hoc test. *$P < 0.05$, **$P < 0.01$ and ***$P < 0.001$.

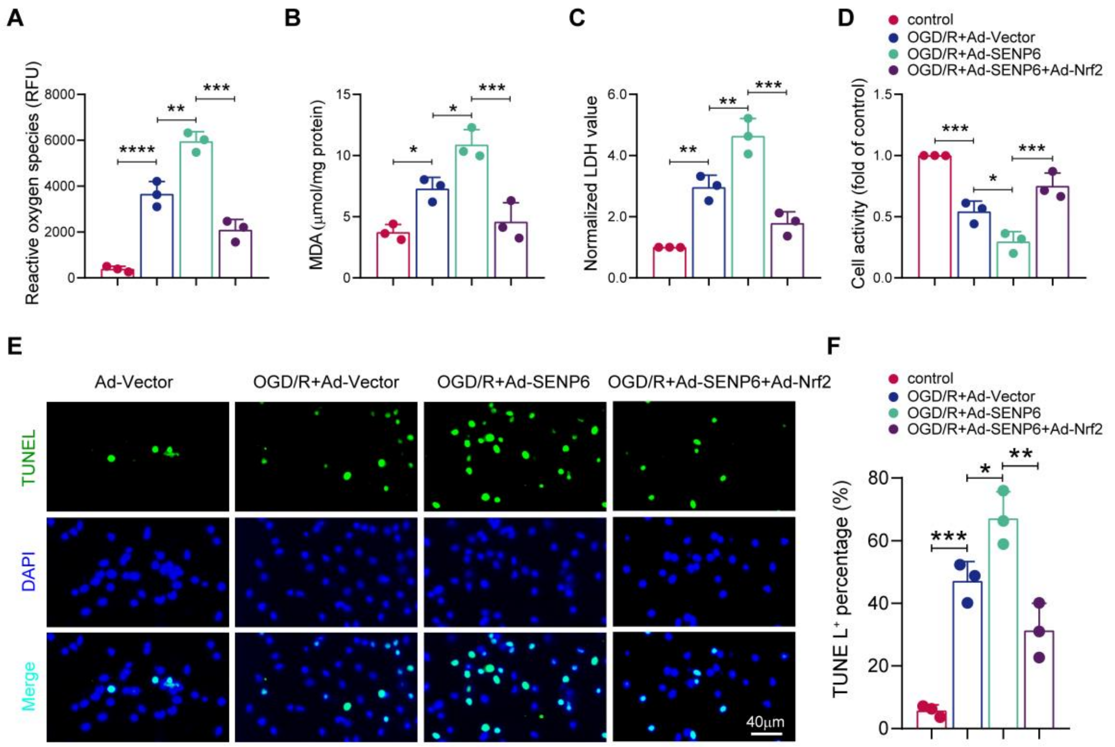

Fig. S5 Overexpression of Nrf2 reversed SENP6-iduced oxidative stress damage and neurotoxicity after ischemic stroke. Primary cultured neurons were transfected with recombinant adenoviruses encoding vector, SENP6, or together with Nrf2 for 48 hours and then challenged with OGD/R. (**A**) Quantitative analysis of ROS measurements. (**B**) Results showing the level of MDA. (**C**) Colorimetry was applied to determine the LDH released from neurons. (**D**) Neuronal viability was examined by CCK-8 assay. (**E**) TUNEL staining was performed to determine the number of apoptotic cells. Scale bar = 40 μm. (**F**) Statistical analysis of TUNEL-positive cells in (E). Data are expressed as the mean ± SD and quantified by one-way ANOVA followed by Tukey's post hoc test. n = 3; *$P < 0.05$, **$P < 0.01$, ***$P < 0.001$ and ****$P < 0.0001$.

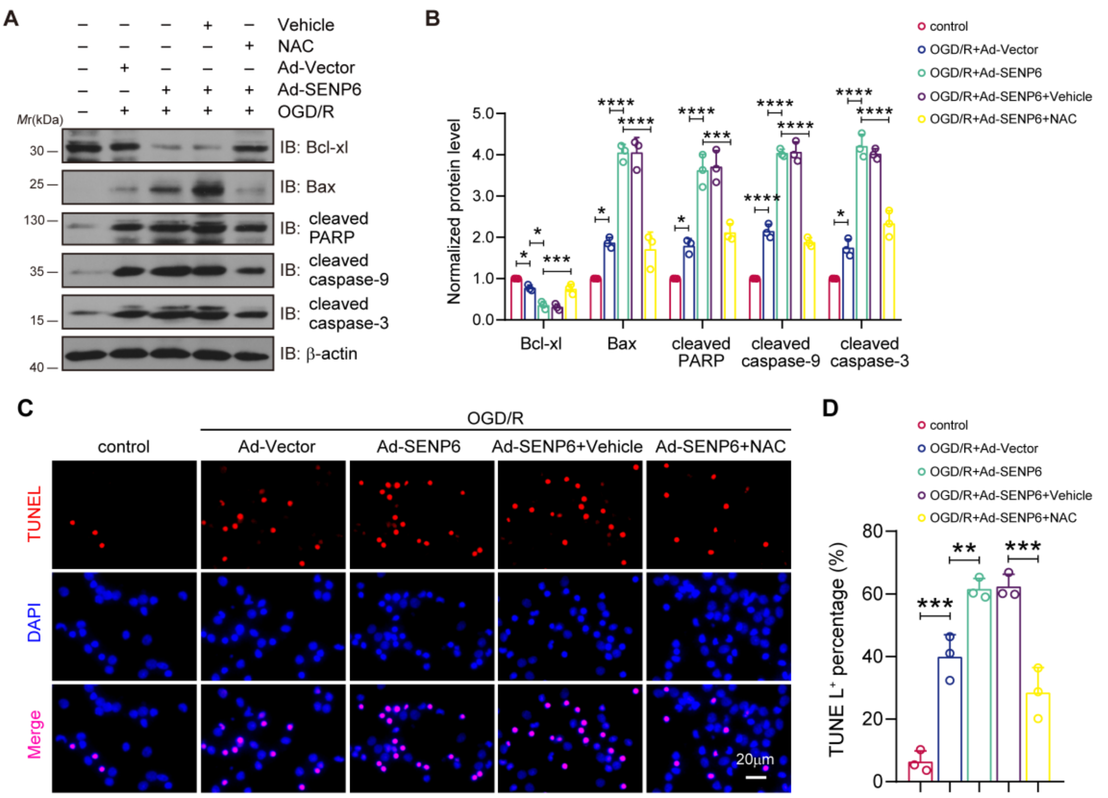

Fig. S6 ***N*-acetyl-L-cysteine treatment revised the promotive role of SENP6 on neuron cell death after OGD/R.** Primary neurons were transfected with recombinant adenoviruses encoding vector, SENP6 for 48 hours and then pretreated with *N*-acetyl-L-cysteine (NAC, 5 mM) for 60 min, after that, the cells were challenged with OGD/R. (**A**) Immunoblot assays was conducted to detected the protein levels of Bcl-xl, Bax, cleaved PARP, cleaved caspase-9 and cleaved caspase-3. (**B**) Quantitative analysis of the indicated protein levels in (**A**). (**C**) TUNEL staining was performed to determine the percentage of apoptotic cells. Scale bar = 20 μm. (**D**) Statistical analysis of TUNEL-positive cells in (C). Data are expressed as the mean ± SD from three independent experiments and quantified by one-way ANOVA followed by Tukey's post hoc test. *$P < 0.05$, **$P < 0.01$, ***$P < 0.001$ and ****$P < 0.0001$.

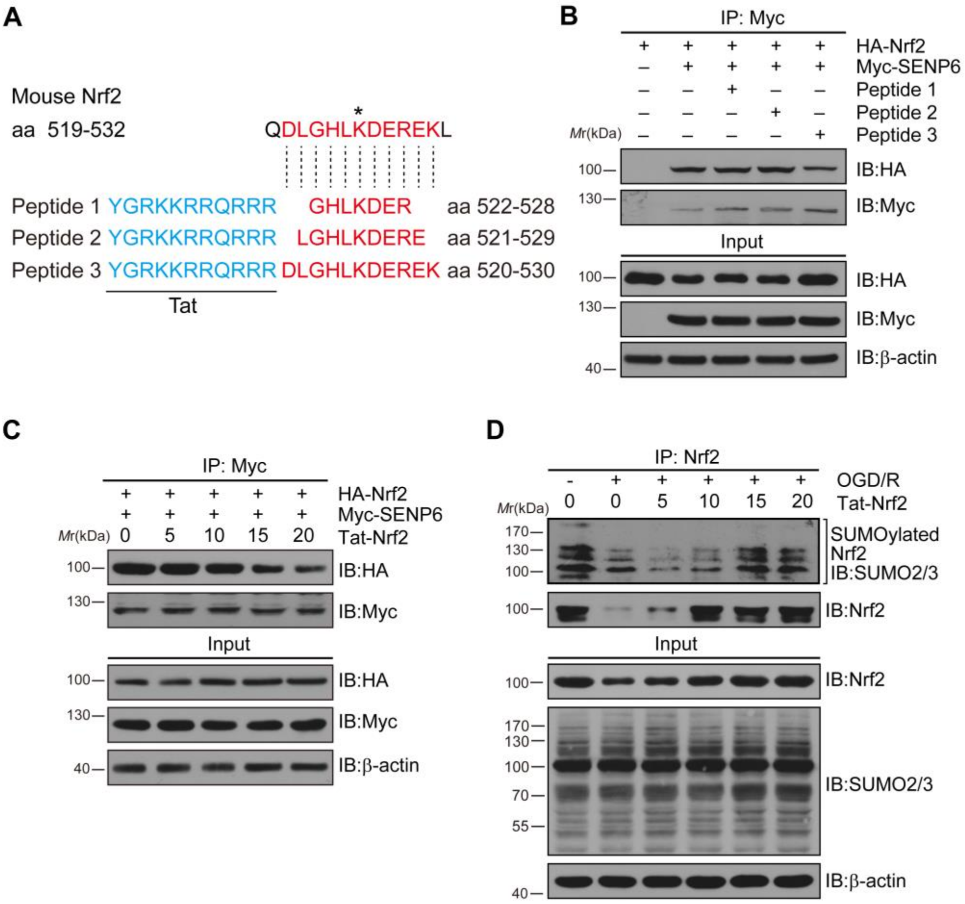

Fig. S7 Tat-Nrf2 peptide treatment effectively blocked the SENP6-Nrf2 interaction and upregulated the SUMOylation level of Nrf2 in vitro. (**A**) Schematic illustration of the peptide design is indicated by an asterisk. (**B**) HEK293T cells were cotransfected with HA-Nrf2 and Flag-SENP6 and then treated with peptide 1, peptide 2, or peptide 3. Co-IP was used to analyze the interaction levels of Nrf2 and SENP6. (**C**) HEK293T cells were cotransfected with HA-Nrf2 and Flag-SENP6 and then treated with Tat-Nrf2 peptide (peptide 3) at the indicated doses for another 3 hours. Co-IP was used to analyze the dose–response effects of the Tat-Nrf2 peptide on the inhibition of the Nrf2-SENP6 interaction. (**D**) Primary cultured neurons were subjected to OGD/R and then treated with Tat-Nrf2 peptide at the indicated doses for another 3 hours. Co-IP was used to analyze the dose–response effects of the Tat-Nrf2 peptide on the levels of Nrf2 SUMOylation. Data are representative of three independent experiments.

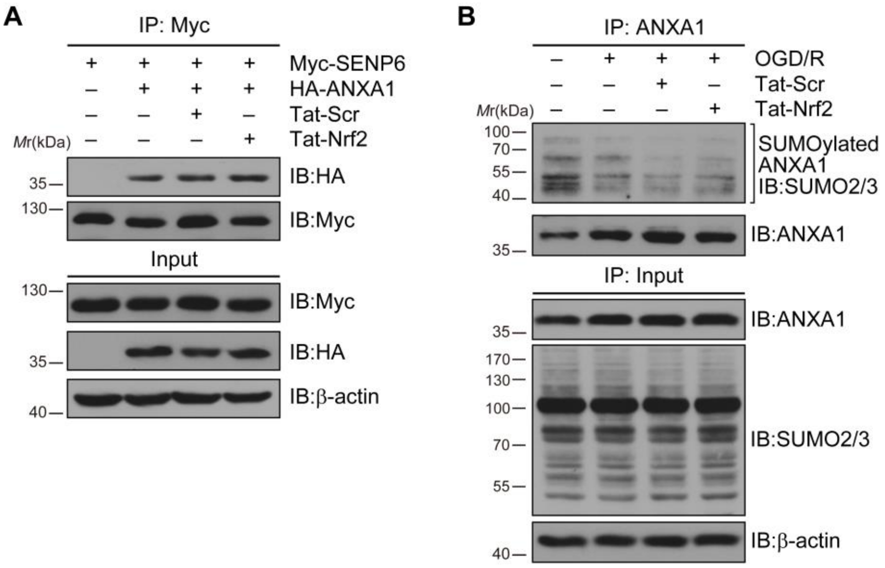

Fig. S8 Tat-Nrf2 peptide has little effect on the SENP6-ANXA1 interaction and the SUMOylation level of ANXA1. (**A**) HEK293T cells were cotransfected with HA-ANXA1 and Flag-SENP6 and then treated with Tat-Nrf2 peptide. Co-IP was used to analyze the interaction levels of ANXA1 and SENP6. (**B**) Primary cultured neurons were subjected to OGD/R and then treated with Tat-Nrf2 peptide at the indicated doses for another 3 hours. Co-IP was used to analyze the effects of the Tat-Nrf2 peptide on the levels of ANXA1 SUMOylation. Data are representative of three independent experiments.

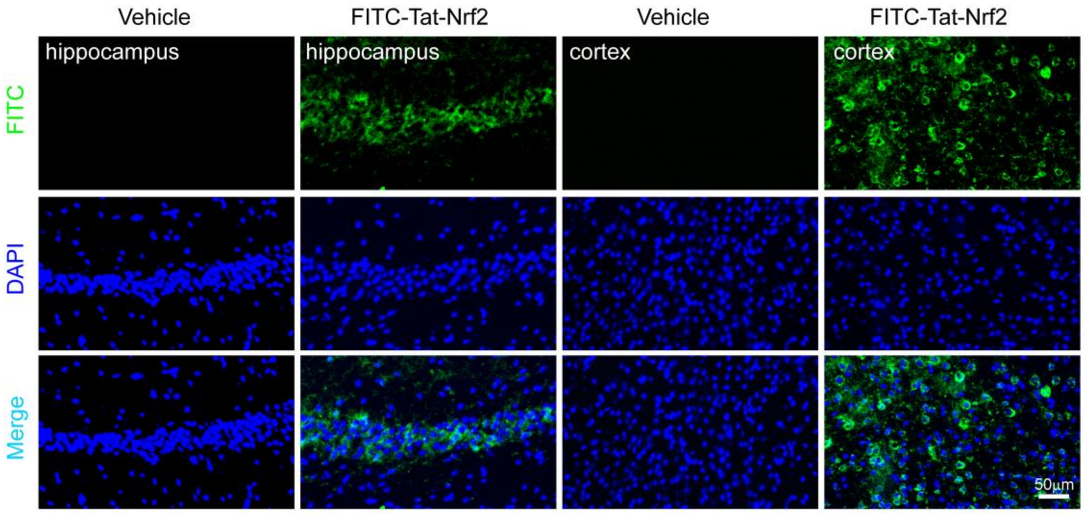

Fig. S9 Tat-Nrf2 peptide was efficiently infused into the hippocampus and cerebral cortex regions. Penetration of FITC-Tat-Nrf2 into hippocampal and cortical tissues in mouse brain after tail vein injection following ischemic stroke. FITC was directly detected on nonfixed tissue slices under fluorescence microscopy. Scale bars, 50 μm.

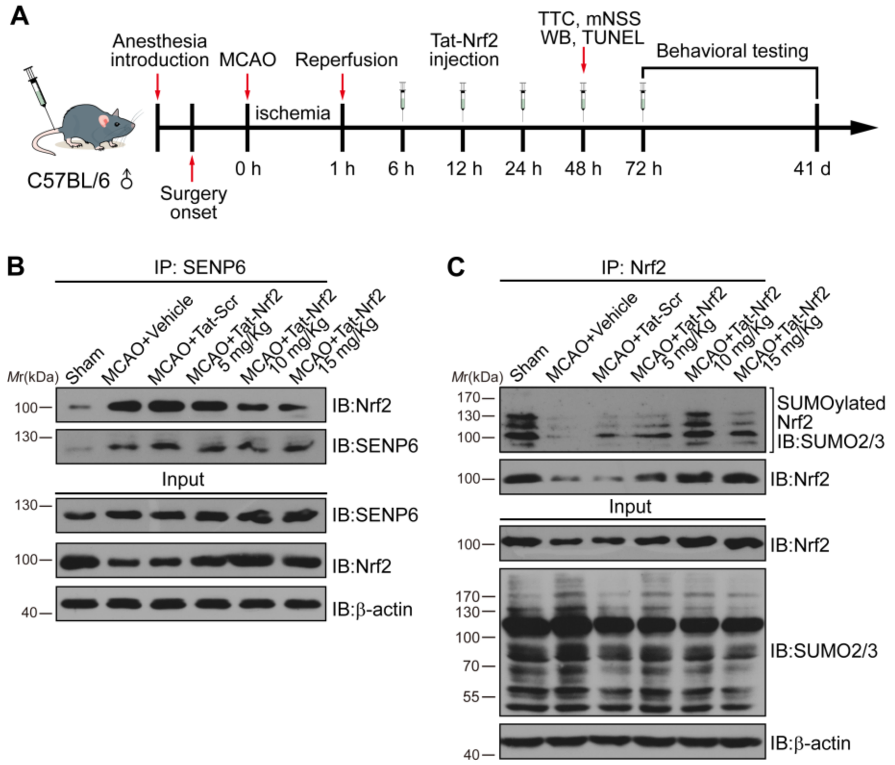

Fig. S10 Tat-Nrf2 peptide treatment effectively blocked the SENP6-Nrf2 interaction and upregulated the SUMOylation level of Nrf2 in mice after ischemic stroke. (**A**) Schematic diagram of the experimental procedure. The animals were intraperitoneally injected with Tat-Scr or Tat-Nrf2 peptides at the indicated dose for 3 days. The mice were subjected to behavioral and histological studies at the indicated time points. (**B**) Co-IP was conducted to analyze the interaction of Nrf2-SENP6 in the ischemic penumbra of brain tissue from mice injected with Tat-Nrf2 or scrambled peptides (10 mg/kg per day) for 3 days. (**C**) Mice were injected with Tat-Nrf2 or scrambled peptides (10 mg/kg per day) for 3 days, and mouse brain homogenates were extracted and the lysates were used for IP with anti-Nrf2 antibody, followed by western blot analysis with anti-SUMO2/3 antibody to detect the SUMOylated band. Data are representative of three independent experiments.

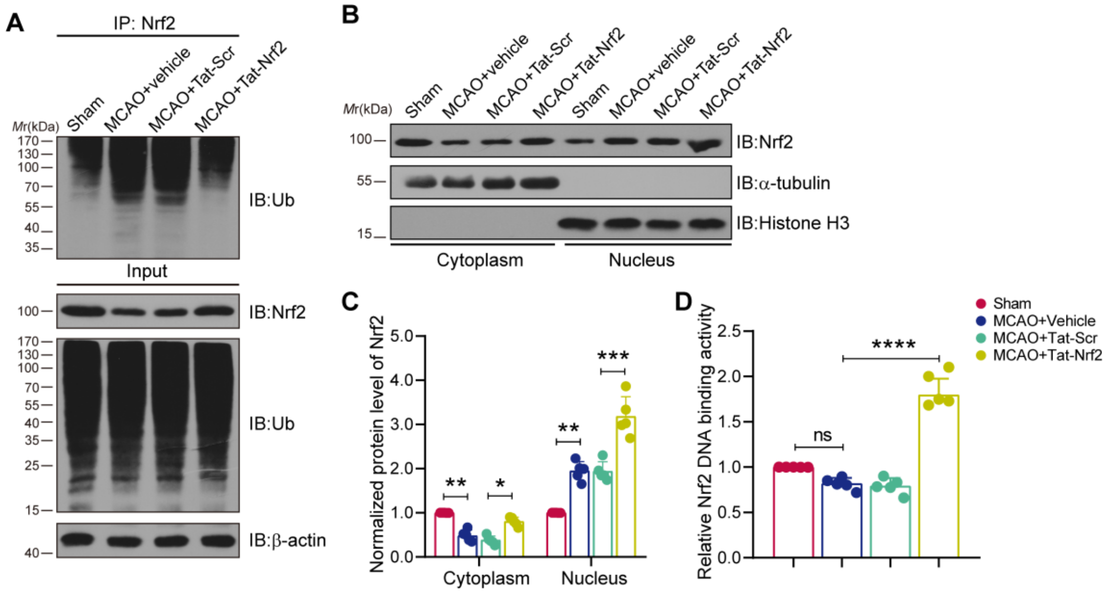

Fig. S11 Tat-Nrf2 peptide treatment decreased the ubiquitination level and promoted the nucleus translocation of Nrf2 in mice after ischemic stroke. (**A**) Immunoprecipitation was conducted to analyze the levels of Nrf2 ubiquitination in the ischemic penumbra of brain tissue from mice injected with Tat-Nrf2 or scrambled peptides (10 mg/kg per day) for 3 days. (**B**) Immunoblot analysis was applied to determine the levels of Nrf2 in cytoplasmic and nuclear extracts. (**C**) Quantification of the immunoblot analysis data shown in (**B**). (**D**) The DNA binding activity of Nrf2 in the ischemic penumbra of brain tissue from mice was determined by an ELISA-based (Trans-AM) method. Data are shown as the mean ± SD and quantified by one-way ANOVA followed by Tukey's post hoc test. n = 3; ns: no significant difference; *$P < 0.05$, **$P < 0.01$, ***$P < 0.001$ and ****$P < 0.0001$.

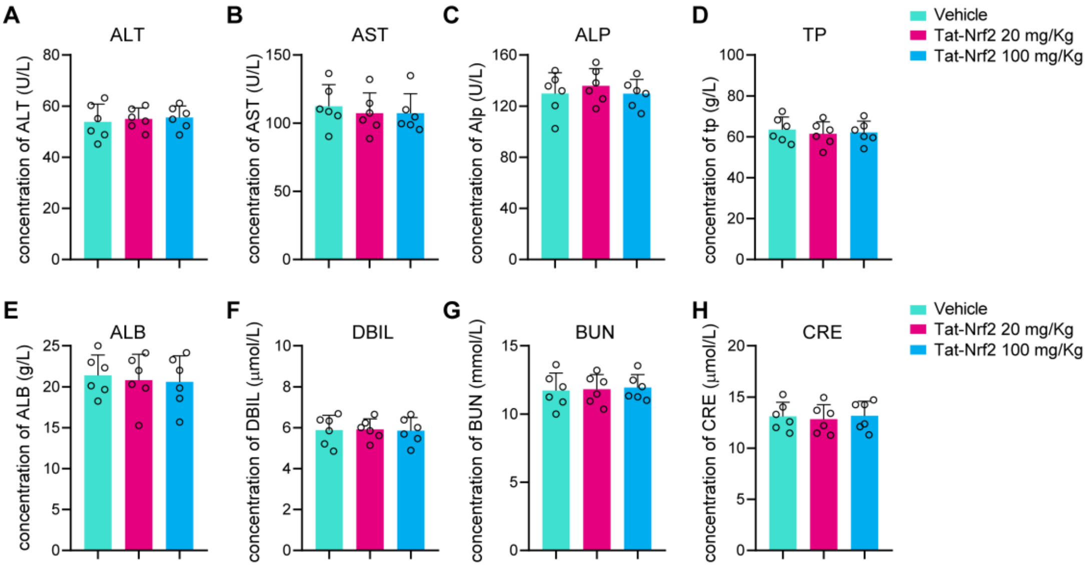

Fig. S12 Safety profile of Tat-Nrf2 peptide. Assays of ALT, AST, ALP, TP, ALB, DBIL, BUN, and CRE levels in the plasma of mice treated with Tat-Nrf2 (i.v.) at a concentration of 20 mg/kg or 100 mg/kg daily for continuous 7 days. ALT, alanine aminotransferase; AST, aspartate aminotransferase; ALP, alkaline phosphatase; TP, total protein; ALB, albumin; DBIL, direct bilirubin; BUN, blood urea nitrogen, and CRE, creatinine. The data are expressed as the mean ± SD. n = 6 mice per group.

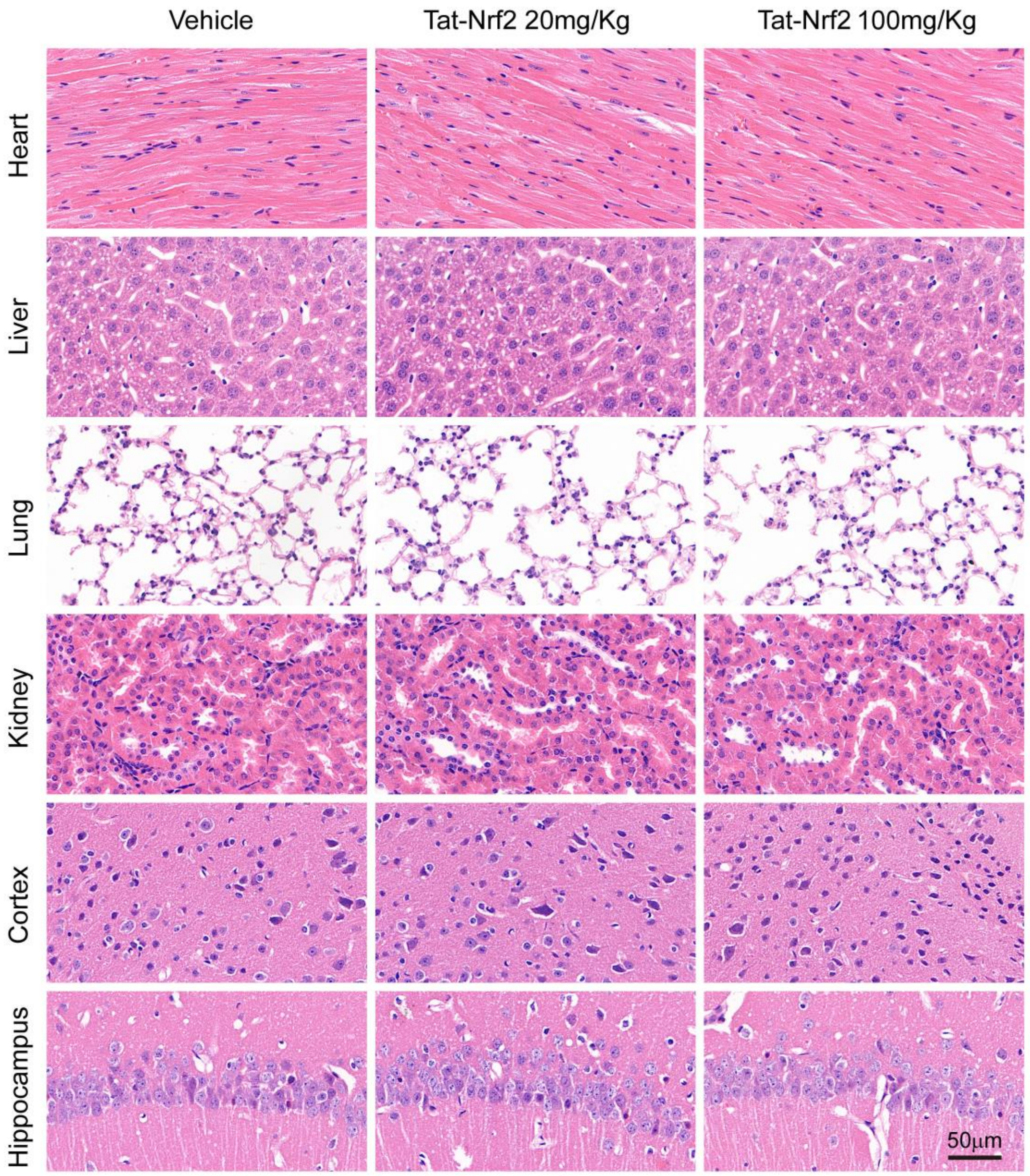

Fig. S13 HE staining images showing the morphology of heart, lung, liver, kidney, and brain in mice. Mice received either Tat-Nrf2 at a concentration of 20 mg/kg, 100 mg/kg, or a similar volume of vehicle daily for 7 days. n = 6 mice per group.

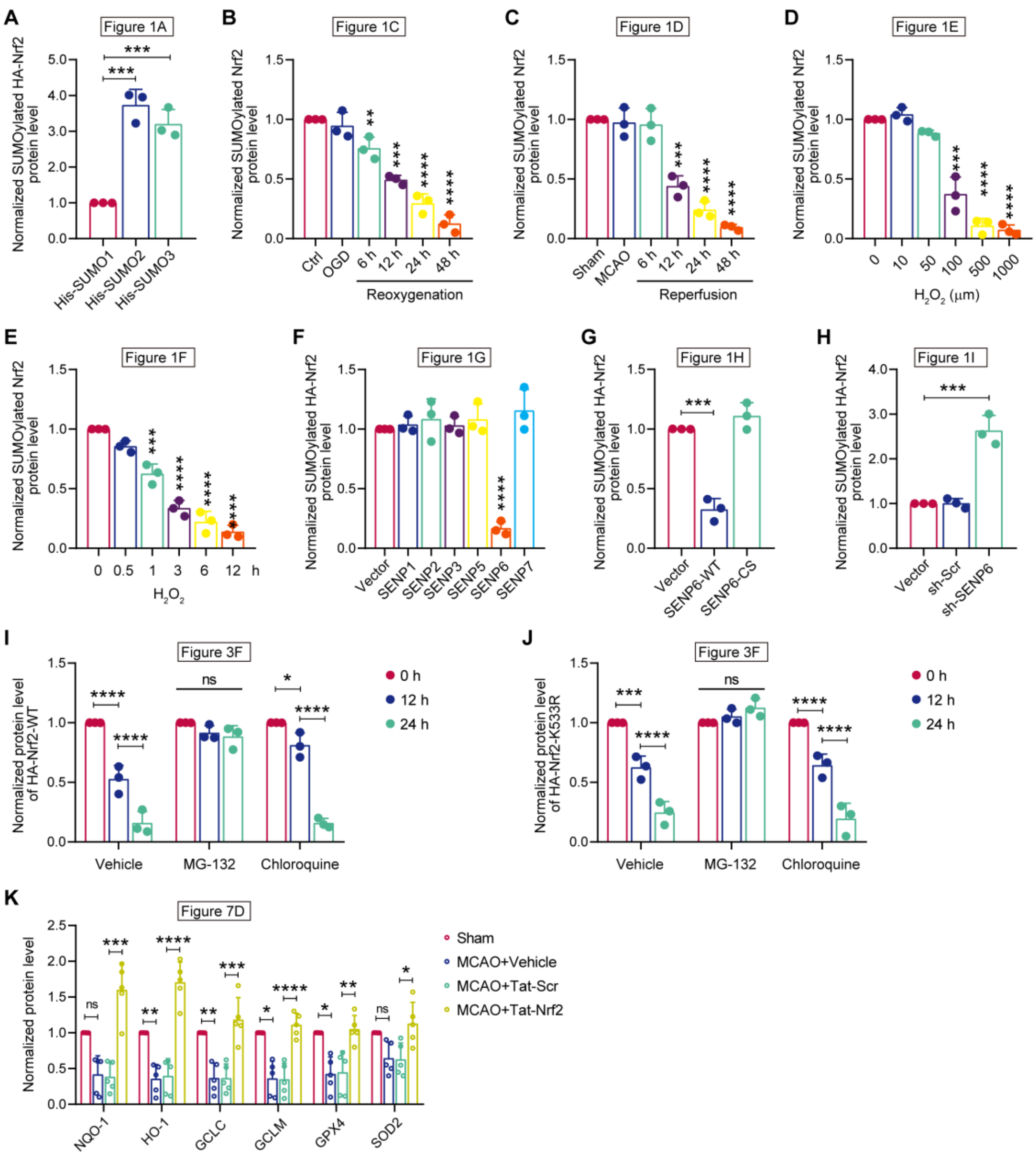

Fig. S14 Quantification of the western blotting data. (**A**) The quantification analysis of the immunoblots of Fig. 1A. Normalized SUMOylated-HA-Nrf2 to corresponding loading control is summarized for three independent experiments. (**B-E**) The quantification analysis of immunoblots of Fig. 1C-F. Normalized SUMOylated-Nrf2 to corresponding loading control is summarized for three independent experiments. (**F-H**) The quantification analysis of immunoblots of Fig. 1G-I. Normalized SUMOylated-HA-Nrf2 to corresponding loading control is summarized for three independent experiments. (**I**, **J**) The quantification analysis of immunoblots of Fig. 3F. Normalized HA-Nrf2 to corresponding loading control are summarized for three independent experiments. (**K**) The quantification analysis of immunoblots of Fig. 7D. Normalized NQO-1, HO-1, GCLC, GCLM, GPX4, SOD2 to corresponding loading control are summarized for three independent experiments. Statistical difference

in panel A-H were determined using one-way ANOVA followed by Dunnett's post hoc test, and all others were analysed by two-way ANOVA followed by Tukey's post hoc test. Data are presented as mean ± SD. ns for $P > 0.05$, *$P < 0.05$, **$P < 0.01$, ***$P < 0.001$ and ****$P < 0.0001$, compared with the control group.

Table S1. Antibodies employed in this study.

Antibody	Species	Type	IB	IF	Source	Identifier
HA	Mouse	Mono-	1:1000		Santa Cruz	sc-7392
Flag	Mouse	Mono-	1:2000		Santa Cruz	sc-166355
His	Rabbit	Poly-	1:1000		Sigma-Aldrich	SAB1306085
GFP	Mouse	Mono-	1:2000		Santa Cruz	sc-9996
Myc	Mouse	Mono-	1:1000		Santa Cruz	sc-40
ANXA1	Mouse	Mono-	1:1000		Santa Cruz	sc-12740
SUMO-2/3	Rabbit	Mono-	1:1000		Cell Signaling	#4971
β-actin	Mouse	Mono-	1:1000		Santa Cruz	sc-47778
Nrf2	Mouse	Mono-	1:1000		Santa Cruz	sc-365949
Nrf2	Rabbit	Poly-		1:200	Proteintech	16396-1-AP
SENP6	Mouse	Mono-	1:1000	1:100	Santa Cruz	sc-100585
Cul3	Rabbit	Poly-	1:1000		Proteintech	10981-2-AP
NQO1	Rabbit	Mono-	1:1000		Cell Signaling	#3187
HO-1	Rabbit	Mono-	1:1000		Cell Signaling	#26416
GCLC	Rabbit	Poly-	1:1000		Proteintech	14241-1-AP
GCLM	Rabbit	Poly-	1:1000		Proteintech	12601-1-AP
GPX4	Rabbit	Mono-	1:1000		Cell Signaling	#59735
SOD2	Mouse	Mono-	1:1000		Cell Signaling	#13141
Bcl-xl	Rabbit	Mono-	1:1000		Cell Signaling	#2764
Bax	Rabbit	Mono-	1:1000		Cell Signaling	#5023
cleaved caspase-3	Rabbit	Mono-	1:1000		Cell Signaling	#9664
cleaved caspase-9	Rabbit	Mono-	1:1000		Cell Signaling	#20750
cleaved PARP	Rabbit	Mono-	1:1000		Cell Signaling	#5625
α-tubulin	Mouse	Mono-	1:2000		Santa Cruz	sc-8035
Histone H3	Rabbit	Mono-	1:2000		Cell Signaling	#4499

Abbreviations: IB, Immunoblotting; IF, Immunofluorescence.

Table S2. Primers used in this study.

Primer name	Primer sequences (5'- 3')

	Forward	Reverse
**Quantitative RT-PCR primers**		
Nqo-1	GCGAGAAGAGCCCTGATTGT	TCAGCTCACCTGTGATGTCATT
Ho-1	CGCAACAAGCAGAACCCAGTC	ACCTCGTGGAGACGCTTTACATAG
Gpx1	ACACCGAGATGAACGATCTG	ATGTACTTGGGGTCGGTCAT
Gpx4	CCTCTGCTGCAAGAGCCTCCC	CTTATCCAGGCAGACCATGTGC
Gclc	GGAGGCGATGTTCTTGAGAC	GGGTGCTTGTTTATGGCTTC
Gclm	AGTTGCACAGCTGGACTCTG	TCGGGTCATTGTGAGTCAGT
Sod1	AAGCGGTGAACCAGTTGTGT	CTGTAAGCGACCTTGCTCCT
Sod2	AACTCAGGTCGCTCTTCAGC	CTGTAAGCGACCTTGCTCCT
Prdx1	AATGCAAAAATTGGGTATCCTGC	CCGTGGGACACACAAAAGTAAA
Gp6d	CACAGTGGACGACATCCGAAA	AGCTACATAGGAATTACGGGCAA
β-actin	TTCGTTGCCGGTCCACACCC	GCTTTGCACATGCCGGAGCC
